# The N-terminal substrate specificity of the SurE peptide cyclase†

**DOI:** 10.1039/d2ob01061e

**Published:** 2022-09-05

**Authors:** Asif Fazal, Jake Wheeler, Michael E. Webb, Ryan F. Seipke

**Affiliations:** Faculty of Biological Sciences, University of Leeds Leeds LS2 9JT UK r.seipke@leeds.ac.uk; Astbury Centre for Structural Molecular Biology, University of Leeds Leeds LS2 9JT UK; School of Chemistry, University of Leeds Leeds LS2 9JT UK

## Abstract

SurE is a standalone peptide cyclase essential for the production of surugamide antibiotics. Although SurE catalyses the cyclisation of varied nonribosomal peptides *in vivo*, its substrate specificity is poorly understood. To address this issue, an on-resin SurE cyclisation assay was developed and in combination with SNAC thioesters and kinetic measurements was used to define the chemical space of the N-terminal substrate residue.

## Abstract 2

The N-terminal substrate specificity of the SurE peptide cyclase was elucidated using a combination of on-resin biomemtic substrates and conventional SNAC thioesters.

## Graphical abstract

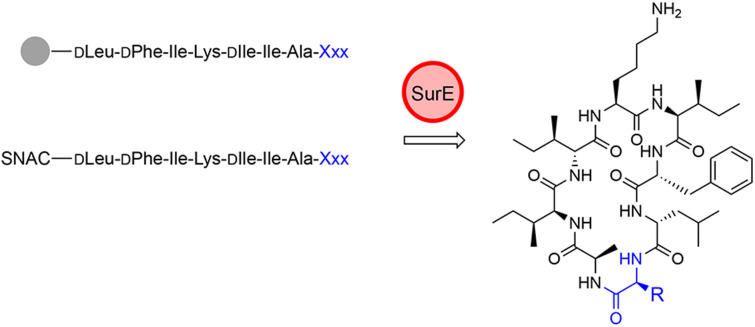

Surugamides A–E are cyclic octapeptide antibiotics produced by terrestrial and marine *Streptomyces* species (Table 1).^1^ While assembly of the peptide scaffold follows canonical nonribosomal peptide biosynthesis logic,^2^ cyclisation of the terminal peptidyl carrier protein (PCP)-bound intermediate is catalysed by a standalone cyclase named SurE, which bears similarity to penicillin-binding proteins.^3^ SurE has been the focus of several recent investigations aimed at understanding its enzymology with a longer-term view of exploiting the novel cyclase to enhance chemical synthesis of cyclic peptide antibiotics and other high-value compounds.^3–7^ These studies revealed that SurE can cyclise *N*-acetylcysteamine (SNAC) thioester substrates designed to mimic their linkage to the PCP 4′-phosphopantetheinyl group. *In vitro* assays with SNAC substrates established that SurE can utilise substrates with changes to ‘internal’ amino acid residues and that the C-terminal (PCP-bound) amino acid residue must be in the d-configuration.^3,5,7^ Despite these advances in understanding, a more complete characterisation of SurE is required for its future biotechnological exploitation.

**Table 1:** Structures of surugamides

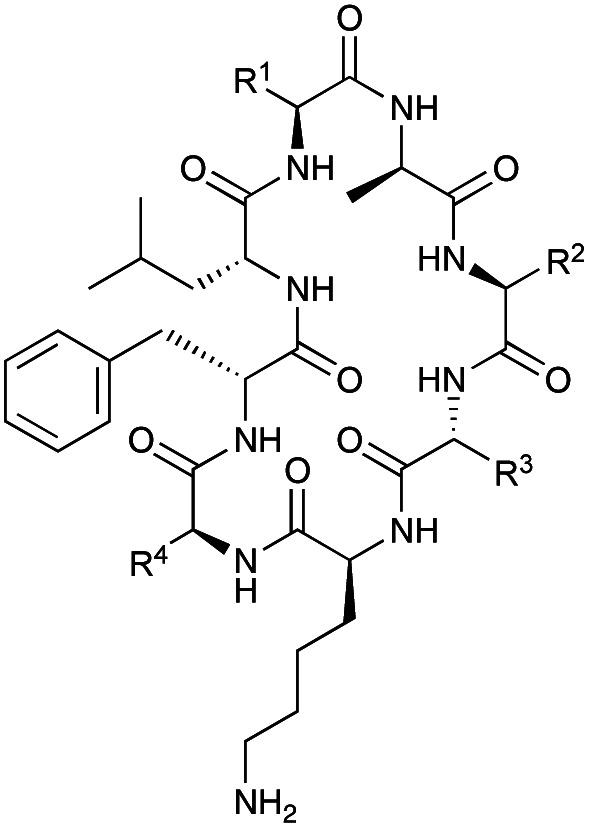				
	R^1^	R^2^	R^3^	R^4^
Surugamide A (1)	Ile	d-Ile	Ile	Ile
Surugamide B (2)	Ile	d-Val	Ile	Ile
Surugamide C (3)	Val	d-Ile	Ile	Ile
Surugamide D (4)	Ile	d-Ile	Val	Ile
Surugamide E (5)	Ile	d-Ile	Ile	Val

Although SNAC substrates are facile to synthesise, they require purification by (semi)-preparative TLC or HPLC, which often precludes use of SNACs for large-scale substrate screening studies. An alternative and more rapid strategy is to analyse substrate specificity on-resin. Such a biomimetic approach exploits intuitive parallels between solid-phase peptide synthesis (SPPS) and NRPS biosynthetic logic in which the growing peptide is tethered to a physical support. This concept was established ∼20 years ago for an excised thioesterase domain in which peptides were tethered to polyethylene glycol amide (PEGA) resin *via* a phosphopantetheine-like linker with an oxo-ester linkage.^8^ The aim of this study was therefore to evaluate the suitability of biomimetic PEGA-peptides for expedited characterisation of SurE. Here we establish that SurE can utilise PEGA-peptides as substrates and in combination with SNAC thioesters and kinetic measurements define the chemical space of the N-terminal substrate residue.

A biomimetic linker was installed on the free amine of PEGA resin after three successive coupling reactions with suberic acid, β-alanine and ethanolamine (Fig. 1).^8^ Unlike the 4′-phosphopantetheinyl group of PCP domains, the synthetic linker terminates in a primary alcohol instead of a thiol; the latter was avoided because of its instability in subsequent steps of SPPS. Next, we synthesised PEGA-surugamide A and a variant harbouring Leu instead of d-Leu at the C-terminus as an established non-cyclisable substrate control.^3,7^ Whilst benchmarking the stability of these PEGA-peptide substrates, it became apparent that simple hydrolysis of peptides off the biomimetic linker can occur (Fig. S1–S4†). Nevertheless, enough test substrate remained intact such that SurE enzyme activity could be assessed in our routine *in vitro* cyclisation assays. As expected, when incubated with PEGA-surugamide A, SurE catalysed the formation of 1, whereas only a trace amount of cyclic product from spontaneous cyclisation was detected in reactions omitting the enzyme or reactions with the Leu-containing non-substrate control (Fig. 1, and Table 2). Thus, PEGA-peptide libraries offer a simplified workflow for evaluating putative substrates for SurE and other peptide cyclases.

**Fig. 1:**
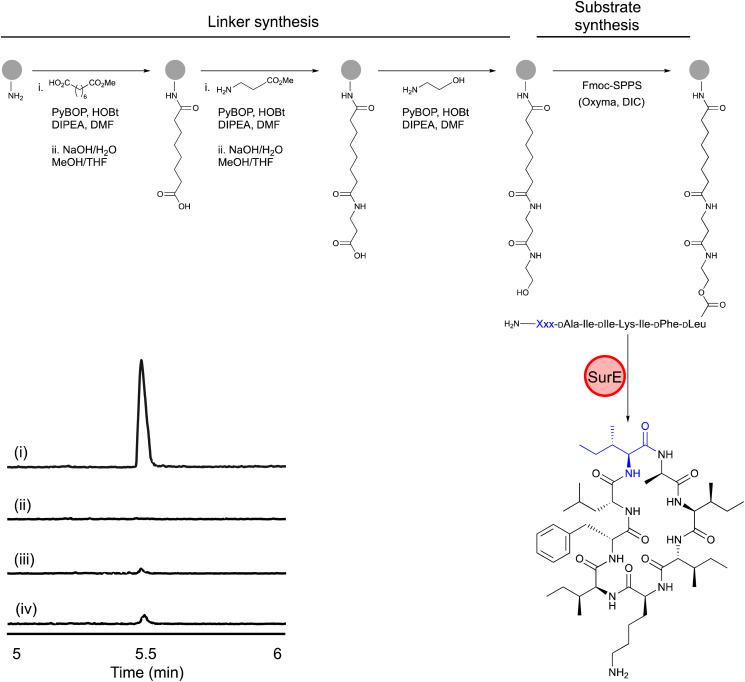
Synthesis of PEGA-peptides and their cyclisation by SurE. (A) Synthetic scheme for PEGA-peptides made in this study; (B) LC-HRMS analysis of SurE cyclisation of PEGA-surugamide A and a non-cyclisable variant harbouring Leu at the C-terminus. Reaction mixture for PEGA-surugamide A reaction mixture with (i) and without SurE (ii). Reaction mixture with the C-terminal Leu PEGA-surugamide A with (iii) and without SurE (iv). The *m*/*z* corresponding to the [M + H]^1+^ ion derived from 1 (C_48_H_81_N_9_O_8_) is shown. The intensity scale is 1 × 10^6^. Grey circles denote PEGA resin; SPPS, solid-phase peptide synthesis; the blue-coloured Xxx and Ile residue denote the N-terminal residue of the substrate.

**Table 2:** SurE cyclisation of PEGA and SNAC substrates

N-Terminal residue	C-Terminal residue	PEGA substrate	SNAC substrate^a^
Ala	d-Leu	Yes	Yes
Arg	d-Leu	No	n.d.
Asn	d-Leu	No	n.d.
Asp	d-Leu	No	n.d.
Cys	d-Leu	No	n.d.
Gln	d-Leu	No	No
Glu	d-Leu	No	n.d.
Gly	d-Leu	Yes	Yes
His	d-Leu	No	n.d.
Leu	d-Leu	Yes	Yes
Ile	d-Leu	Yes	Yes
Ile	Leu	No	No
Lys	d-Leu	Yes	Yes
Met	d-Leu	No	n.d.
Orn	d-Leu	Yes	Yes
Phe	d-Leu	No	n.d.
Pro	d-Leu	No	n.d.
Ser	d-Leu	No	n.d.
Thr	d-Leu	Yes	Yes
Trp	d-Leu	Yes	Yes
Tyr	d-Leu	No	n.d.
Val	d-Leu	Yes	Yes

a n.d. = not determined.

Next, we used our PEGA-based strategy to interrogate amino acid residues tolerated at the N-terminal position of surugamide A. We generated a library of PEGA-peptides in which the native N-terminal Ile residue was replaced with the 19 remaining proteinogenic amino acids or Orn. Each PEGA-peptide was then individually used as a test substrate for SurE.

The resulting data is summarised in Table 2 and depicted in Fig. S5–S24.† Interpretation of these data was aided by previous reports indicating that N-Val and N-Trp, but not N-Pro variants are substrates for SurE.^5,7^ Thus, we considered a PEGA-peptide to be a SurE substrate if the MS2 fragmentation pattern was consistent with the monoisotopic mass of the anticipated cyclised product with a parent ion count >10^6^. This framework allowed us to determine PEGA-peptides harbouring Ala, Gly, Leu, Lys, Orn, Thr and Val at the N-terminus were substrates for SurE, while PEGA-peptides with N-terminal Asn, Asp, Arg, Cys, Gln, Glu, His, Met, Phe, Pro, Ser, Trp and Tyr residues were not (Table 2, and Fig. S5–S24†). Although we detected a monoisotopic mass consistent with the cyclised Trp-variant, the associated MS2 spectra did not permit unambiguous confirmation of the cyclised product (Fig. S22†). This was unexpected, given a SNAC thioester Trp-variant of surugamide B was previously shown to be a SurE substrate,^5^ a finding which we verify and discuss below.

In practical terms, we treated the above PEGA-peptide assays as a traffic light screen to identify putative substrates for SurE. Thus, the seven substrates identified above, and the native variant (Ile) were synthesised as traditional SNAC thioesters and purified for more detailed analyses. We also synthesised the SNAC thioesters of the N-terminal Gln variant as a representative non-SurE substrate control to further validate our PEGA-peptide screen and the N-terminal Trp variant to further investigate its utilisation by SurE. The ability of SurE to utilise the above SNAC thioesters was assessed by LC-HRMS. These data are summarised in Table 2 and displayed in Fig. S25–S42.† As anticipated, SurE does not cyclise the non-substrate control harbouring an N-terminal Gln residue, but the expected cyclic products were detected when the cyclase was incubated with all of the other SNAC substrates tested, including the N-terminal Trp variant (Table 2). Analysis of mass spectrometry data for cyclisation assays with SNAC and PEGA substrates indicates the cyclised variant with Trp at the N-terminus does not ionise well, which may be the underlying cause for why it was not initially classified as an accepted substrate in our initial screen. We therefore repeated the SurE cyclisation assay for PEGA-Trp-surugamide and positive (PEGA-Orn-surugamide) and negative (PEGA-Gln-surugamide) controls, but a higher substrate concentration. The resulting data (Fig. S45–S48†) allowed us to unambiguously establish that the PEGA-Trp-surugamide is accepted by SurE.

In order to provide a more detailed characterisation of SurE substrate utilisation, we developed a colorimetric assay based on the widely used thiol-reactive compound 5,5′-dithiobis(2-nitrobenzoic acid) (DTNB). Reaction of DNTB with free thiol results in stoichiometric production of the yellow 2-nitro-5-thiobenzoate anion (TNB), which enabled us to measure the amount of *N*-acetylcysteamine released in our assays and therefore compare enzymatic activity of SurE on substrates evaluated in this study. Cyclisation assays were carried out at substrate concentrations of 100 and 200 μM to compare initial catalysis rates, and these data are summarised in Fig. 2, Fig. S43 and Table S1.† SurE displayed good levels of activity towards SNAC-substrates with Gly, Ala, Thr, Lys, or Orn at the N-terminus, retaining between 70 and 115% of the activity observed for the native substrate under these reaction conditions. The N-Val and N-Leu variants were also utilised efficiently by SurE; ∼1.5× improvements to initial rates were observed for both substrates. The similarity to the Ile-containing native surugamide A peptide likely means a structurally similar enzyme–substrate complex is formed with these N-terminal variants. Notably the N-Gln variant was not effectively turned over by SurE, confirming its nature as a non-cyclisable substrate. The Trp-containing variant, however, was utilised particularly effectively, displaying a substantial increase in rate of catalysis. This is in accordance with a previous report testing an N-Trp variant, showing the substrate was particularly amenable to SurE-catalysed cyclisation.^5^

**Fig. 2:**
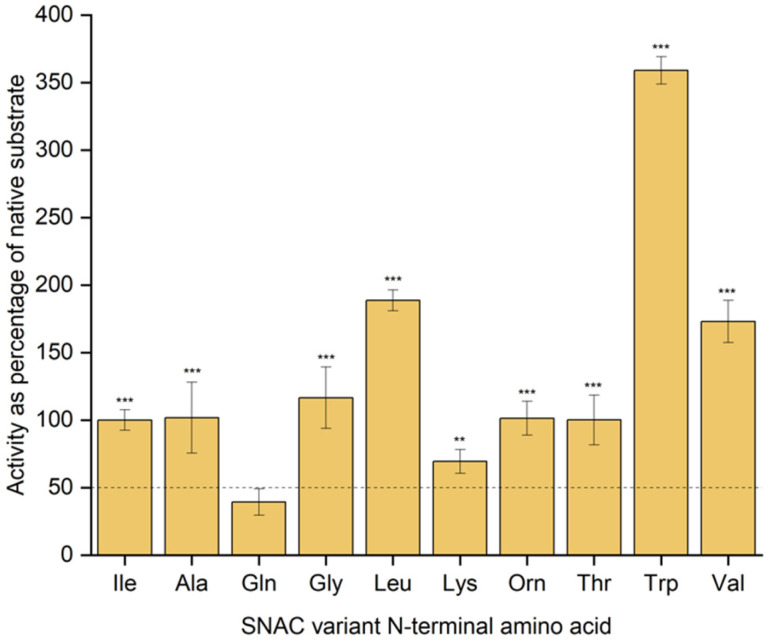
Enzymatic activity of SurE with SNAC-surugamide variants as indicated. Calculated mean initial rates for each substrate assay, carried out in triplicate and at a substrate concentration of 200 μM, are shown for each substrate as a percentage of the initial rate observed for the native (Ile) surugamide peptide. Pairwise *t* tests were performed for each substrate with the Gln-containing, putative non-substrate (***: *p* value <0.001). The horizontal line at *y* = 50 represents a threshold above which mean initial rate yields a significant *p* value (assuming zero error in any measurements and 95% confidence limit).

Taken together, these data indicate that SurE shows promiscuity with the N-terminal residue of its substrate and that changes to this position have a relatively minor influence to the formation of the peptidyl-*O*-SurE intermediate involving the C-terminus, but likely a more significant impact to substrate turnover.

## Conclusions

In summary, we have developed an on-resin biomimetic approach to assay substrate utilisation by standalone peptide cyclase enzymes, which negates the requirement for time consuming purification steps commonly leading to yield loss. As a proof-of-concept, we used our approach to scan the N-terminus of the SurE peptide substrate and together with conventional SNAC thioesters and kinetic analyses determined that the SurE cyclase only possesses relatively modest specificity for the N-terminus of its substrate. Our study is the first comprehensive analysis of substrate utilisation by SurE-family cyclases and is an important step towards developing bioengineering strategies to exploit this unique family of enzymes.

## Author contributions

Conceptualization (AF, MEW, RFS), formal analysis (AF), funding acquisition (RFS, MEW), resources (JW), supervision (RFS, MEW), visualisation (AF, RFS), writing – original draft (AF, RFS), writing – reviewing & editing (AF, JW, MEW, RFS).

## Conflicts of interest

There are no conflicts to declare.

## Supplementary Material

OB-020-D2OB01061E-s001
