# Supplementary material for: The N-terminal substrate specificity of the SurE peptide cyclase
Source: Org Biomol Chem. 2022 Sep 5;20(36):7232–5. doi: 10.1039/d2ob01061e (PMC9491159; doi:10.1039/d2ob01061e)
Supplement: OB-020-D2OB01061E-s001 [file OB-020-D2OB01061E-s001.pdf]

Electronic Supplementary Material (ESI) for Organic & Biomolecular Chemistry.
This journal is © The Royal Society of Chemistry 2022

SUPPLEMENTARY INFORMATION

The N-terminal substrate specificity of the SurE peptide cyclase

Asif Fazal[a,b,c], Jake Wheeler[b,c], Michael E. Webb[b,c], Ryan F. Seipke[a,b]*

[a] *Faculty of Biological Sciences, University of Leeds, Leeds, LS2 9JT, UK.*

[b] *Astbury Centre for Structural Molecular Biology, University of Leeds, Leeds, LS2 9JT, UK*

[c] *School of Chemistry, University of Leeds, Leeds, LS2 9JT, UK.*

***Corresponding author:**

Ryan F. Seipke

E-mail: r.seipke@leeds.ac.uk

Table of contents:

Experimental procedures
Figure S1. LC-HRMS analysis of PEGA-surugamide assays
Figure S2. MSMS analysis of peptides produced from PEGA-surugamide assays
Figure S3. LC-HRMS analysis of PEGA-Leu-surugamide assays
Figure S4. MSMS analysis of peptides produced from PEGA-surugamide-Leu assays
Figure S5. LC-HRMS analysis of assays of SurE with all N-terminal variant PEGA-peptides
Figure S6. MSMS analysis of peptides produced from PEGA-Ala-surugamide/SurE assay
Figure S7. MSMS analysis of peptides produced from PEGA-Arg-surugamide/SurE assay
Figure S8. MSMS analysis of peptides produced from PEGA-Asn-surugamide/SurE assay
Figure S9. MSMS analysis of peptides produced from PEGA-Asp-surugamide/SurE assay
Figure S10. MSMS analysis of peptides produced from PEGA-Gln-surugamide/SurE assay
Figure S11. MSMS analysis of peptides produced from PEGA-Glu-surugamide/SurE assay
Figure S12. MSMS analysis of peptides produced from PEGA-Gly-surugamide/SurE assay
Figure S13. MSMS analysis of peptides produced from PEGA-His-surugamide/SurE assay
Figure S14. MSMS analysis of peptides produced from PEGA-Leu-surugamide/SurE assay
Figure S15. MSMS analysis of peptides produced from PEGA-Lys-surugamide/SurE assay
Figure S16. MSMS analysis of peptides produced from PEGA-Met-surugamide/SurE assay
Figure S17. MSMS analysis of peptides produced from PEGA-Orn-surugamide/SurE assay
Figure S18. MSMS analysis of peptides produced from PEGA-Phe-surugamide/SurE assay
Figure S19. MSMS analysis of peptides produced from PEGA-Pro-surugamide/SurE assay
Figure S20. MSMS analysis of peptides produced from PEGA-Ser-surugamide/SurE assay
Figure S21. MSMS analysis of peptides produced from PEGA-Thr-surugamide/SurE assay
Figure S22. MSMS analysis of peptides produced from PEGA-Trp-surugamide/SurE assay
Figure S23. MSMS analysis of peptides produced from PEGA-Tyr-surugamide/SurE assay
Figure S24. MSMS analysis of peptides produced from PEGA-Val-surugamide/SurE assay
Figure S25. LC-HRMS analysis of SNAC-Gln-surugamide A/SurE assays
Figure S26. MSMS analysis of peptides produced from SNAC-Gln-surugamide A/SurE assays
Figure S27. LC-HRMS analysis of SNAC-Ala-surugamide A/SurE assays
Figure S28. MSMS analysis of peptides produced from SNAC-Ala-surugamide A/SurE assays
Figure S29. LC-HRMS analysis of SNAC-Gly-surugamide A/SurE assays
Figure S30. MSMS analysis of peptides produced from SNAC-Gly-surugamide A/SurE assays
Figure S31. LC-HRMS analysis of SNAC-Leu-surugamide A/SurE assays
Figure S32. MSMS analysis of peptides produced from SNAC-Leu-surugamide A/SurE assays
Figure S33. LC-HRMS analysis of SNAC-Lys-surugamide A/SurE assays
Figure S34. MSMS analysis of peptides produced from SNAC-Lys-surugamide A/SurE assays
Figure S35. LC-HRMS analysis of SNAC-Orn-surugamide A/SurE assays
Figure S36. MSMS analysis of peptides produced from SNAC-Orn-surugamide A/SurE assays
Figure S37. LC-HRMS analysis of SNAC-Thr-surugamide A/SurE assays
Figure S38. MSMS analysis of peptides produced from SNAC-Thr-surugamide A/SurE assays
Figure S39. LC-HRMS analysis of SNAC-Trp-surugamide A/SurE assays
Figure S40. MSMS analysis of peptides produced from SNAC-Trp-surugamide A/SurE assays
Figure S41. LC-HRMS analysis of SNAC-Val-surugamide A/SurE assays
Figure S42. MSMS analysis of peptides produced from SNAC-Val-surugamide A/SurE assays
Figure S43. LC-HRMS analysis of SNAC-surugamide A/SurE/DTNB assay
Figure S44. Plot displaying SurE activity on each of the N-terminal SNAC-surugamide variant substrates as a percentage of activity on the native substrate
Table S1. Calculated mean initial rates of activity of SurE with N-terminal SNAC-surugamide variant substrates
Figure S45. LC-HRMS analysis of larger scale PEGA-Gln-surugamide A/SurE assays

Figure S46. LC-HRMS analysis of larger scale PEGA-Orn-surugamide A/SurE assays
Figure S47. LC-HRMS analysis of larger scale PEGA-Trp-surugamide A/SurE assays
Figure S48. MSMS analysis of cyclic peptide produced from the larger scale PEGA-Trp-surugamide A/SurE assay

Materials and methods

Growth media, strains, plasmids and other reagents. *Escherichia coli* strains were propagated on Lennox agar (LA) or broth (LB).[1] Culture media was supplemented with kanamycin (50 µg mL^{-1}). Enzymes were purchased from New England Biolabs unless otherwise stated and oligonucleotides were purchased from Integrated DNA Technologies.

Chemical synthesis of SNAC- and PEGA-linker-surugamide substrates. Linear SNAC-surugamide peptides were synthesized by SPPS and purified using preparative HPLC as described previously.[2] Solid phase PEGA-linked substrates were synthesized by SPPS as according to.[3] PEGA resin was swollen in DCM for at least 30 minutes at room temperature with stirring. To assemble the biomimetic linker, the resin was coupled twice with monomethyl suberic acid using 4.9 equivalents of benzotriazol-1-yloxytripyrrolidinophosphonium hexafluorophosphate (PyBOP), 5 equivalents of hydroxybenzotriazole (HOBt), and 10 equivalents of DIPEA in DMF for two hours with stirring. The ester methyl group was deprotected to leave the free acid, using a base deprotection step of 30 min stirring in tetrahydrofuran (THF)/ MeOH/ 10N NaOH (6: 3: 1), followed by a dilute acid wash. Five equivalents of β-alanine methyl ester were then coupled twice to the resin using the same coupling procedure as used for suberic acid, before base deprotection and a dilute acid wash to leave the free acid. Ethanolamine•HCl (20 equivalents) were coupled to the resin using the same coupling procedure to complete the chemical linker. The first amino acid of the substrate peptide was then coupled to the free hydroxyl of the linker by SPPS using oxyma, DIC, and the amino acid in DMF. The solution was stirred with the resin for 1 hour before elution and deprotection by the series of washes: 3 × 2min DMF, 2 × 5min 20% piperidine, 5 × 1min DMF. Subsequently required amino acids were then coupled to the resin using the same procedure, until the terminal amino acid was deprotected. PEGA resin – linker – peptide substrates were then used without further cleavage or purification in enzyme assays as required.

SurE cyclisation assays with SNAC- and PEGA-linked substrates. $(His)_6$SurE was overproduced and purified from *E. coli* BL21(Gold)DE3 and used in cyclisation assays with SNAC substrates exactly as described previously.[2] Cyclisation assays with PEGA-linked substrates were carried out as follows. In a final reaction volume of 500 µL, PEGA-substrate pre-swollen in methanol was incubated with 10 µM of purified protein in 50 mM HEPES (pH 7.5), in a Biotage Isolute 3 mL column equipped with a single frit, at 25 °C for 5 hours. The solvent was separated from the resin by elution with a vacuum manifold, and the resin was washed with 200 µL of acetonitrile. Reruns of PEGA-surugamide (PEGA-Gln-surugamide A, PEGA-Orn-surugamide A, PEGA-Trp-surugamide A assays were scaled up and carried out on 4x the amount of PEGA-tethered peptide. The larger amount of methanol pre-swollen peptide was incubated with 10 µM of purified protein in 50 mM HEPES (pH 7.5), at 30 °C for 5 hours, before the solvent was separated from the resin by elution with a vacuum manifold. The elutions were analysed directly by injecting 1 uL peptide sample in 0.1% formic acid on to a Vanquish LC system (Thermo Scientific) using a flow rate of 0.3 mL min^{-1}. Peptides were desalted/separated using a Phenomenex Kinetex C18 column (2.6 um particle size, 50 mm x 4.6 mm) held at 30 °C. Starting mobile phase composition was 1 % solvent B (0.1% formic acid in acetonitrile) in A (0.1% formic acid in water) for one minute followed by a gradient elution of 1% B to 95% B in 2.5 min. The column was washed at 95% B for 1 min following be re-equilibration at 1% B for 1.5 mins. Peptides were eluted in to an Orbitrap Exploris 240 mass spectrometer and ionised using electrospray ionisation in positive polarity at 3500 V. Mass measurement used full scan mode resolution of 60000, a *m/z* range of 350-2000. MS/MS selection was based on signal intensity of greater that 5x105 counts and charge state of 1-3. Peptides selected for MS/MS were fragmented in the HCD cell using a relative collision energy of 30 % and measured with a resolution of 15000. LC-HRMS analysis of SNAC-peptide/SurE assays was carried out using a Bruker MaXis Impact II TOF mass

spectrometer equipped with a Dionex UltiMate 3000 UHPLC using conditions previously described.[2]

Kinetic analysis of SNAC substrate cyclisation. Reactions of purified $(His)_6$SurE and SNAC-peptides for kinetic analysis were carried out using the thiol-modifying compound 5,5'-dithiobis(2-nitrobenzoic acid) (DTNB).[4,5] Reactions were set up in 96-well microtitre plates in a total volume of 200 µL and using 50 mM HEPES (pH 7.5), 200 mM NaCl as the assay buffer. Each assay well contained 1 µM of $(His)_6$SurE and SNAC-substrate in a concentration of either 100 or 200 µM, in the presence of DTNB (4 µL of 10 mM stock solution). Controls reactions were also set up and contained either: buffer only; buffer and DTNB only; buffer, DTNB, and substrate only; or buffer, DTNB, and protein only. Assay plates were set up in triplicate and incubated at 30 °C for 15 min inside the chamber of a BMG Labtech Omega plate reader, with absorbance monitoring at λ = 412 nm. Absorbance over time graphs were plotted for each assay condition. Initial rates were calculated by determining the gradient of the line-of-best-fit for the first six data points for all reactions except the Lys variant where the first five data points were used. Calculated average initial rates were compared to the rate observed for the native surugamide A peptide to generate activity as a percentage of WT for each variant substrate. Pairwise, 2-tailed t tests were performed for each substrate with the putative non-substrate (Gln-containing) and p values were generated at the 95% confidence limit.

References

1. T. Kieser, M. J. Bibb, M. J. Buttner, K. F. Chater, D. A. Hopwood, *Practical Streptomyces genetics,* John Innes Foundation, 2000.

2. D. Thankachan, A. Fazal, D. Francis, L. Song, M. E. Webb and R. F. Seipke, *ACS Chem. Biol.,* 2019, **14,** 845–849.

3. R. M. Kohli, C. T. Walsh and M. D. Burkart, *Nature,* 2002, **418,** 658–661.

4. D. Schwarzer, H. D., Mootz, U., Linne, M. A., Marahiel, *Proc. Natl. Acad. Sci. USA,* 2002, **99,** 14083–14089.

5. U., Linne, D., Schwarzer, G. N., Schroeder, M. A., Marahiel, *Eur. J. Biochem.,* 2004, **271,** 1536–1545.

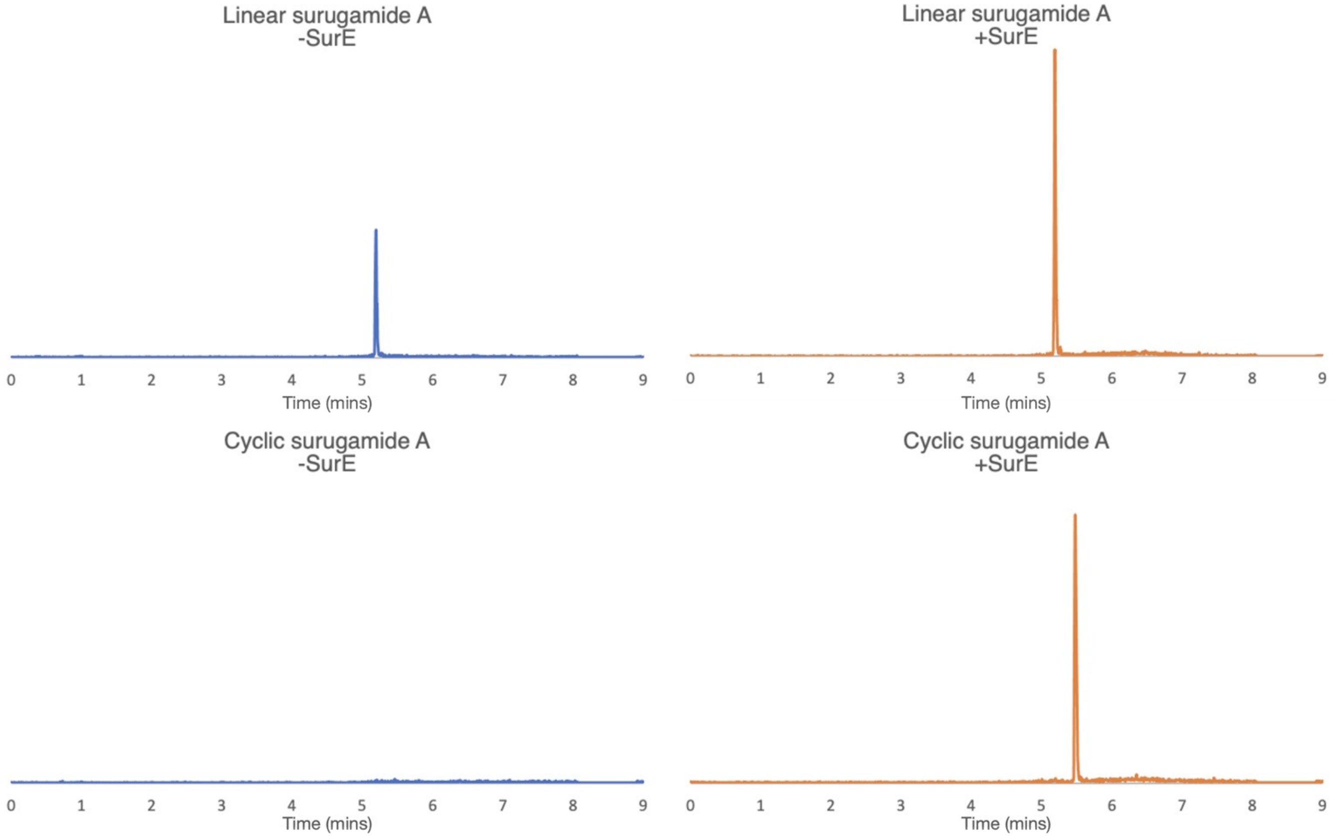

Fig. S1. LC-HRMS analysis of assays involving PEGA-surugamide A in the presence (orange) and absence (blue) of SurE. Extracted ion chromatograms (EICs) are shown for ions corresponding to the *m/z* values of $[M+H]^+$ and $[M+Na]^+$ ions of linear surugamide A ($C_{48}H_{83}N_9O_9$) and surugamide A ($C_{48}H_{81}N_9O_8$). The intensity scale for all linear-surugamide A and surugamide A EICs was 2×10^5.

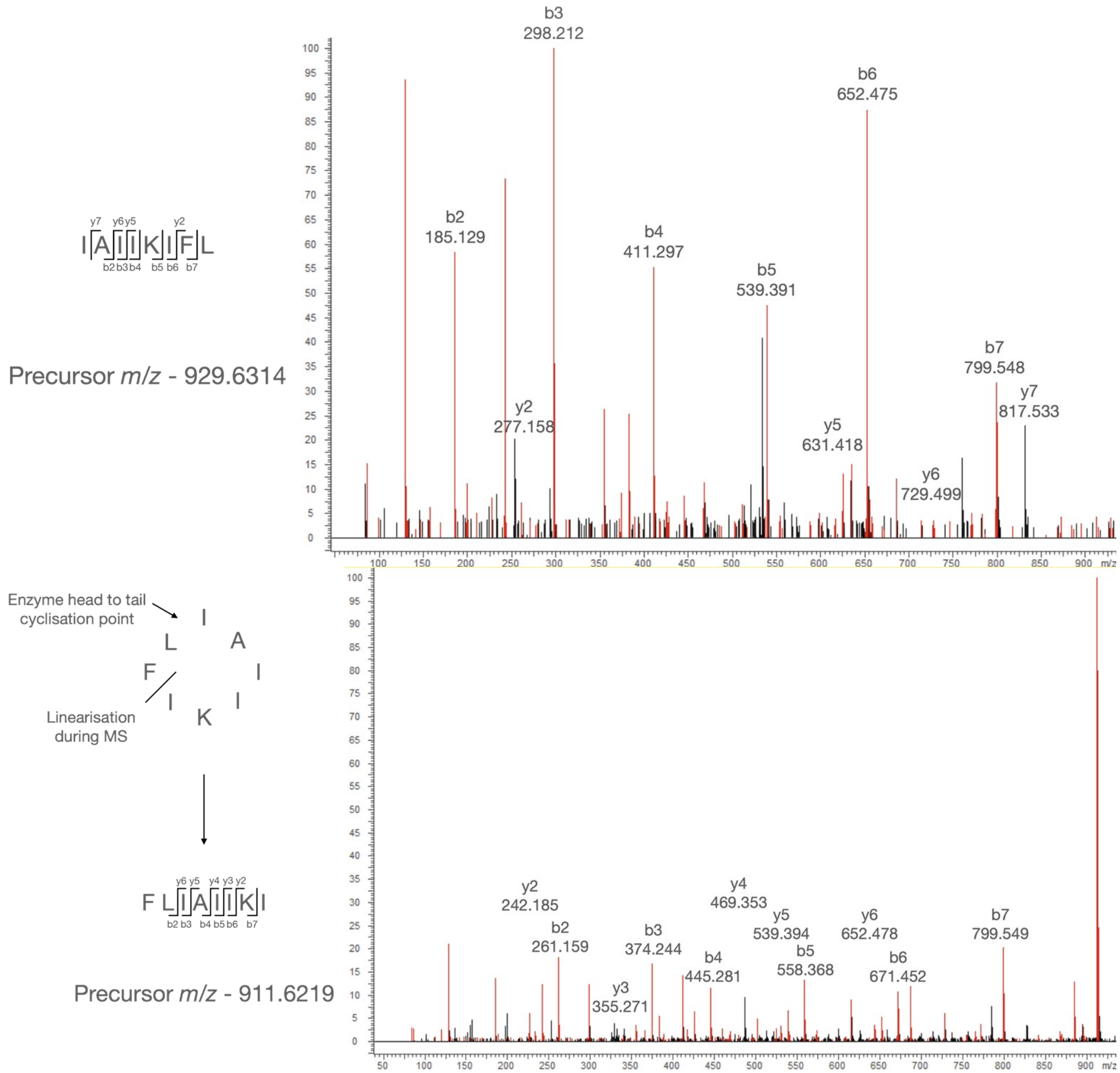

Fig. S2. MSMS analysis of surugamide peptides produced during assay of PEGA-surugamide A with SurE. Analysis of fragment ions produced from linear (top) and cyclic (bottom) forms of surugamide A are shown, with *b* and *y* ions labelled with *m/z* values above the corresponding mass spectral peak. The precursor ion and nature of each of the formed fragment ions are shown on the side of each analysis.

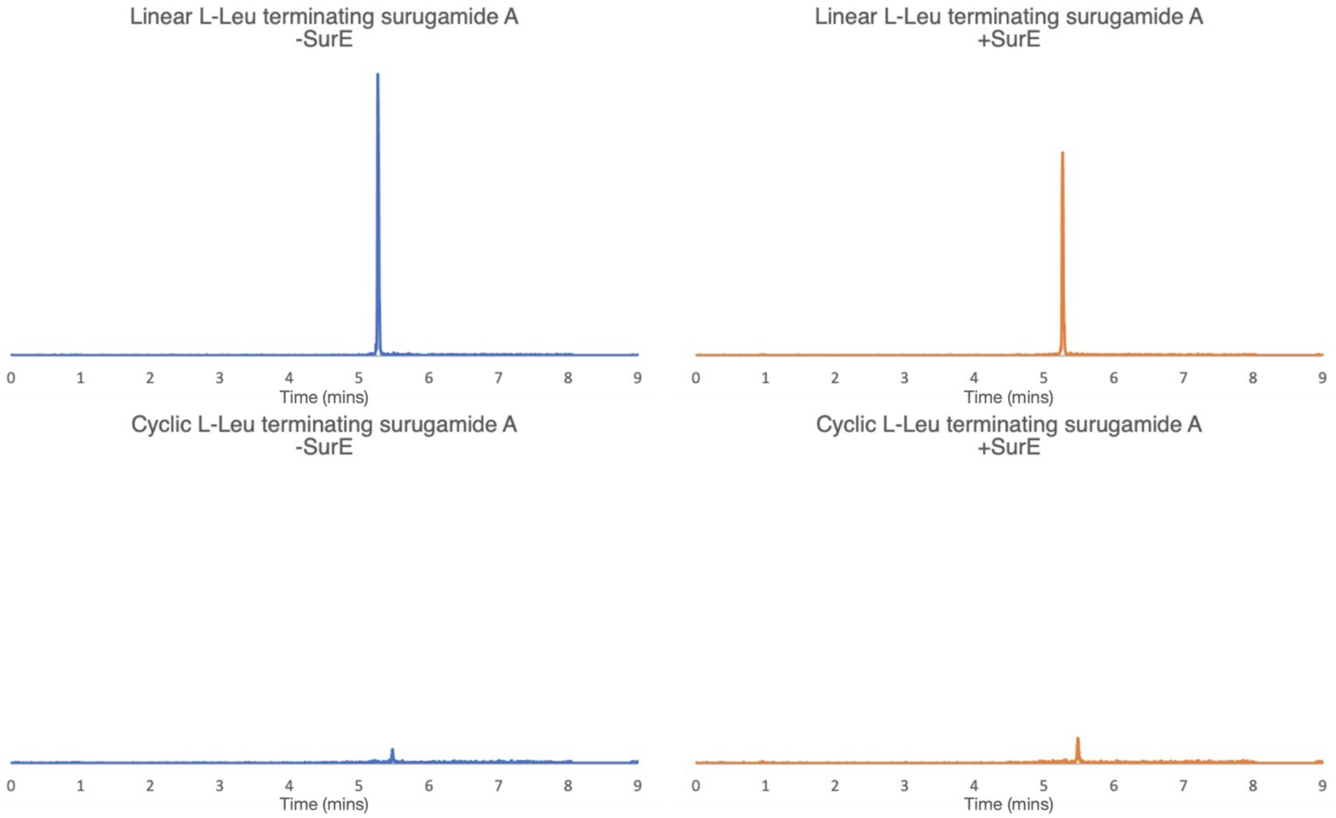

Fig. S3. LC-HRMS analysis of assays involving PEGA-surugamide-Leu in the presence (orange) and absence (blue) of SurE. Extracted ion chromatograms (EICs) are shown for ions corresponding to the *m/z* values of $[M+H]^+$ and $[M+Na]^+$ ions of linear surugamide A ($C_{48}H_{83}N_9O_9$) and surugamide A ($C_{48}H_{81}N_9O_8$). The intensity scale for all linear-Leu-surugamide A EICs was 3×10^5 and 1.8×10^5 for all cyclic Leu-surugamide A EICs.

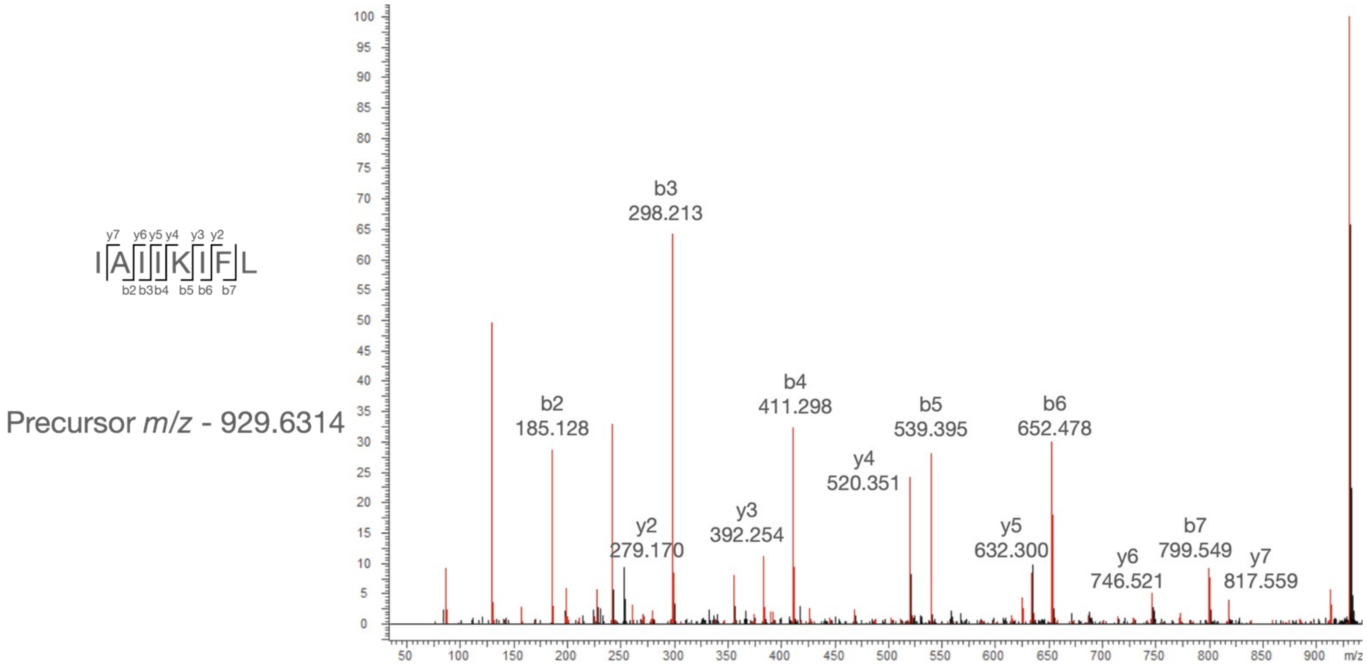

Fig. S4. MSMS analysis of the linear Leu-terminating surugamide A peptide produced during assay of PEGA-surugamide-Leu with SurE. Analysis of fragment ions produced from the linear peptide is shown, with *b* and *y* ions labelled with *m/z* values above the corresponding mass spectral peak. The precursor ion and nature the formed fragment ions are shown on the side of each analysis. No MSMS analysis could be performed on the observed peak for the cyclic peptide due to the low intensity nature of the formed fragments.

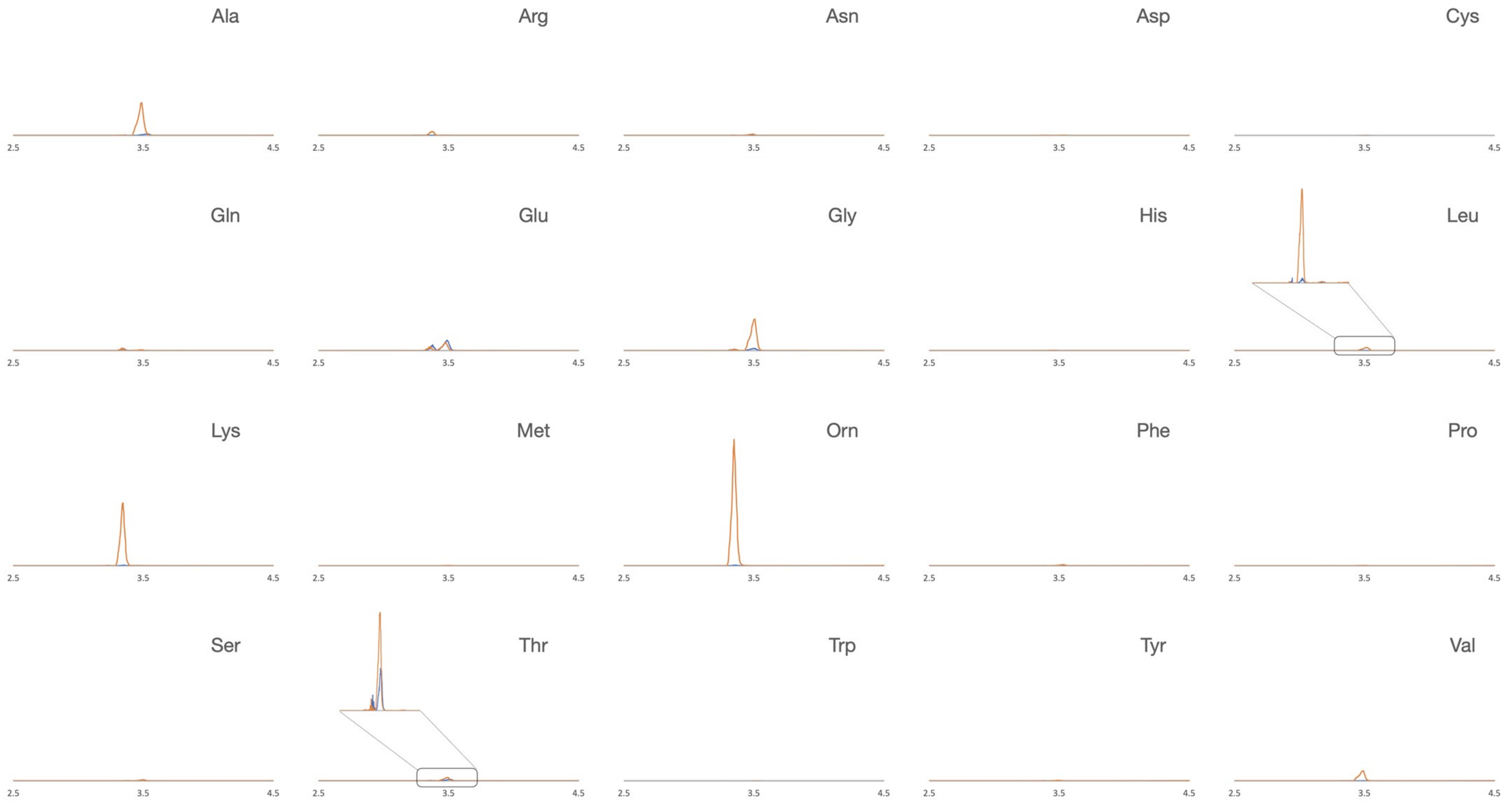

Fig. S5. LC-HRMS analysis of assays of N-terminal variant surugamide peptides appended to PEGA resin with (orange) and without (blue) SurE. The EIC corresponding to the *m/z* of the cyclic peptide in each case is represented. The *y*-axis was set to 8×10^7 for all chromatograms. A zoomed view of the peak-containing region is shown in the case of the Thr- and Leu- N-terminal variant peptides to display the peaks. The *y*-axis was set to 2×10^6 for the graphs in the zoomed sections.

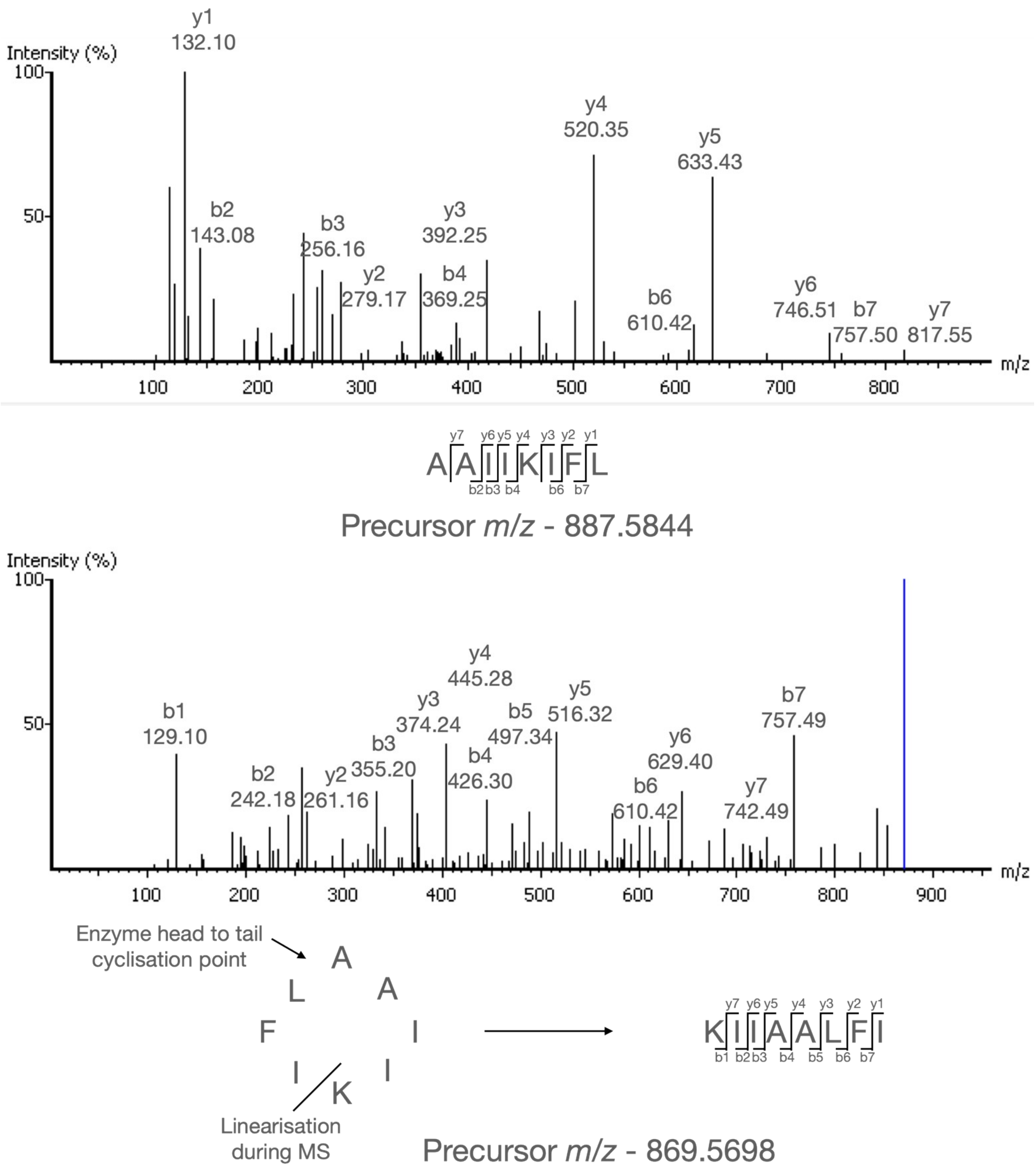

Fig. S6. MSMS analysis of fragment ions produced during assay of PEGA-Ala-surugamide A (the N-terminal Ala variant of surugamide A appended to the PEGA resin) and SurE. Both linear and cyclic peptides were produced. The peaks corresponding to each fragment ion are labelled with the representative *m/z* value. The nature of the precursor peptide is shown below the mass spectrum in each case.

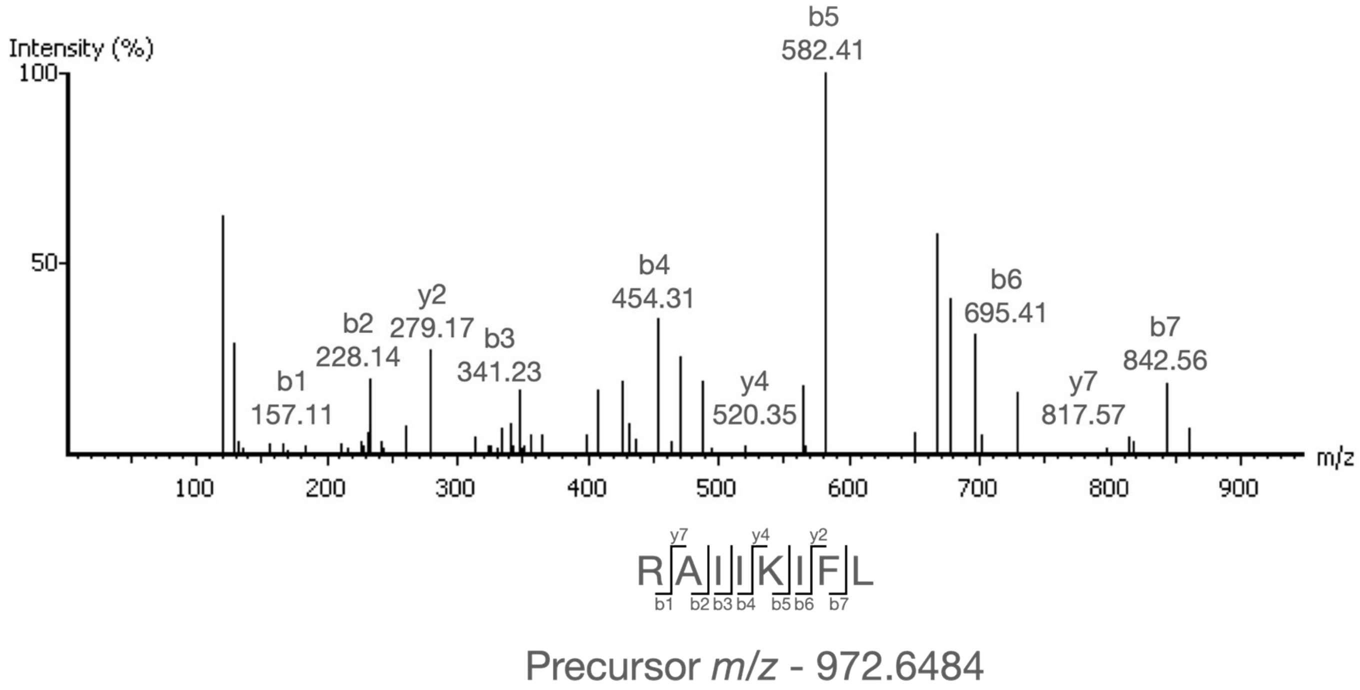

Fig. S7. MSMS analysis of fragment ions produced during assay of PEGA-Arg-surugamide A (the N-terminal Arg variant of surugamide A appended to the PEGA resin) and SurE. Only a linear peptide was produced. The peaks corresponding to each fragment ion are labelled with the representative *m/z* value. The nature of the precursor peptide is shown below the mass spectrum.

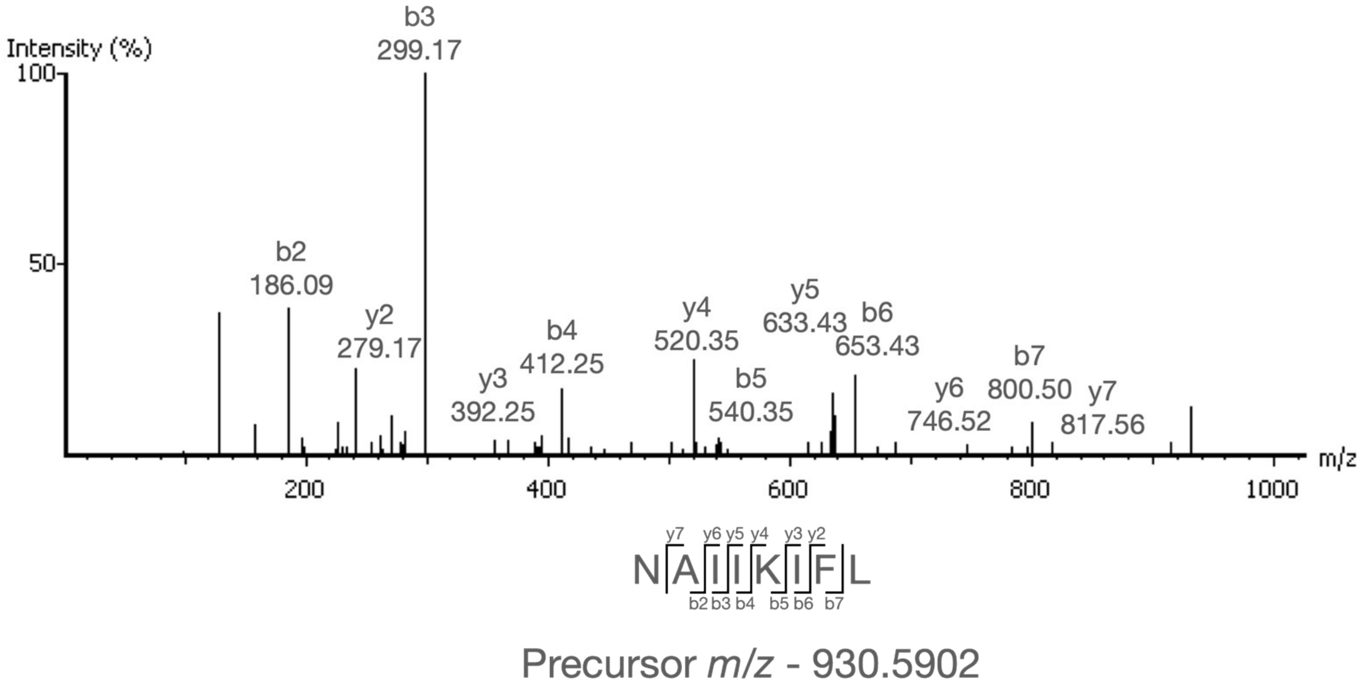

Fig. S8. MSMS analysis of fragment ions produced during assay of PEGA-Asn-surugamide A (the N-terminal Asn variant of surugamide A appended to the PEGA resin) and SurE. Only a linear peptide was produced. The peaks corresponding to each fragment ion are labelled with the representative *m/z* value. The nature of the precursor peptide is shown below the mass spectrum.

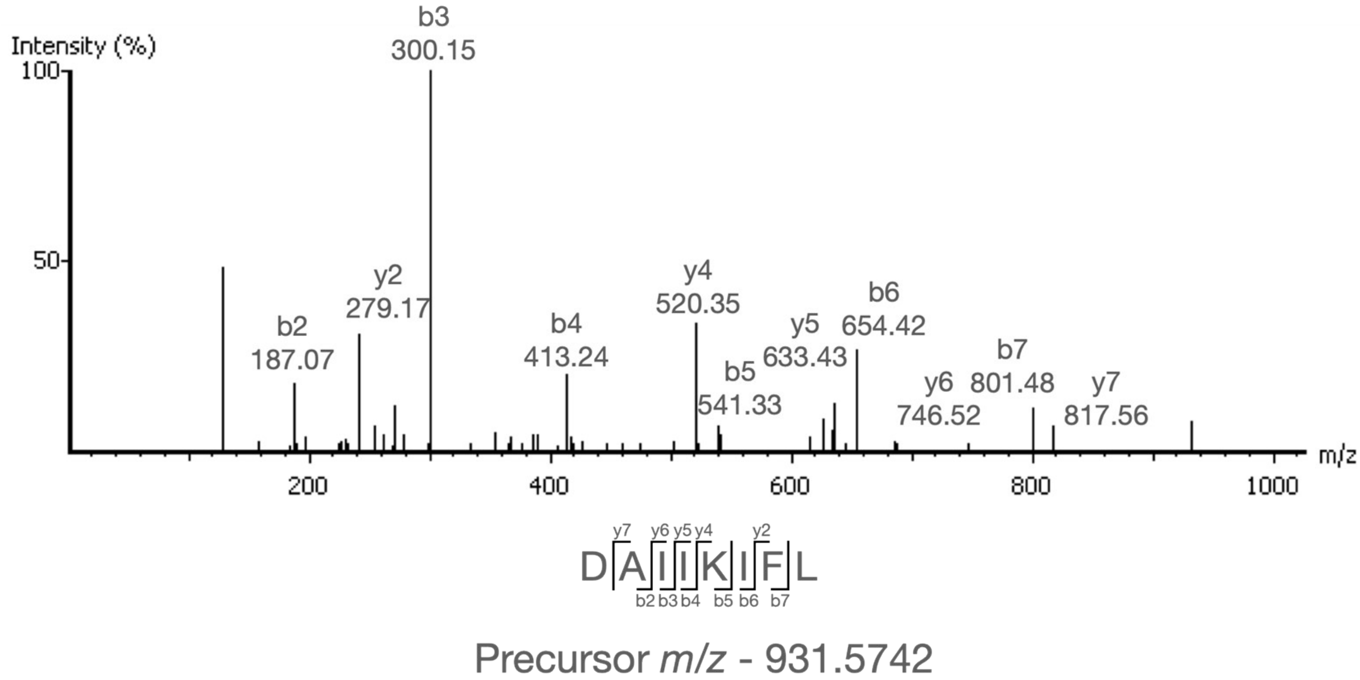

Fig. S9. MSMS analysis of fragment ions produced during assay of PEGA-Asp-surugamide A (the N-terminal Asp variant of surugamide A appended to the PEGA resin) and SurE. Only a linear peptide was produced. The peaks corresponding to each fragment ion are labelled with the representative *m/z* value. The nature of the precursor peptide is shown below the mass spectrum.

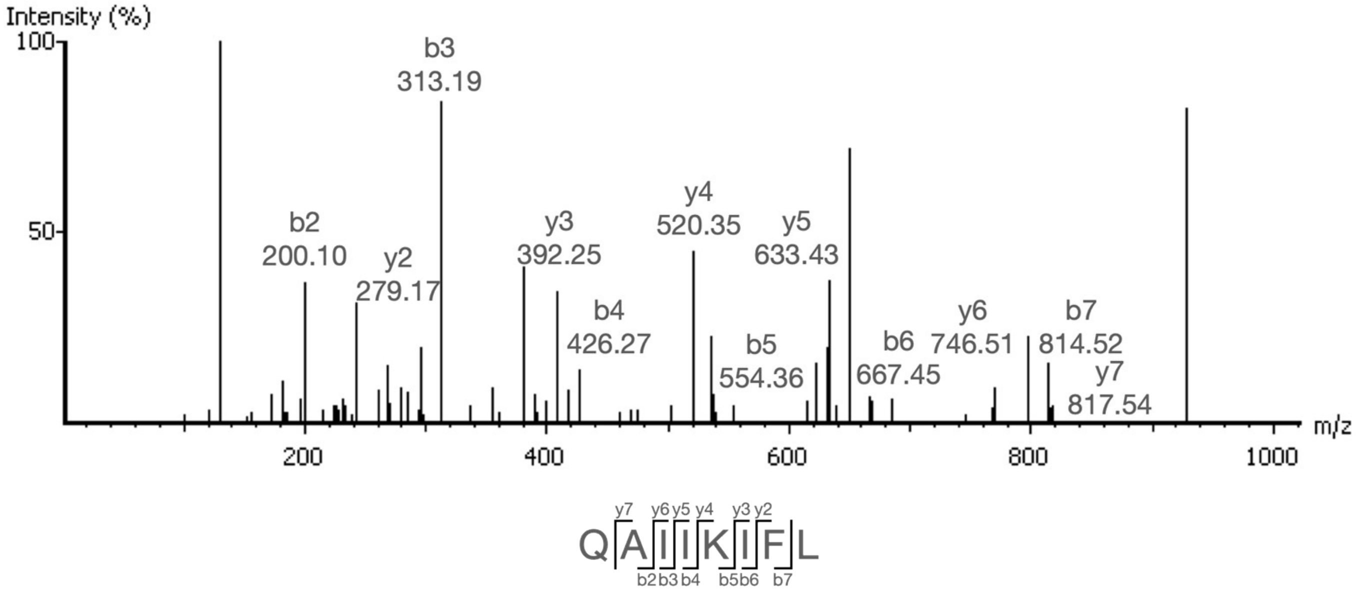

Precursor *m/z* - 944.6058

Fig. S10. MSMS analysis of fragment ions produced during assay of PEGA-Gln-surugamide A (the N-terminal Gln variant of surugamide A appended to the PEGA resin) and SurE. Only a linear peptide was produced. The peaks corresponding to each fragment ion are labelled with the representative *m/z* value. The nature of the precursor peptide is shown below the mass spectrum.

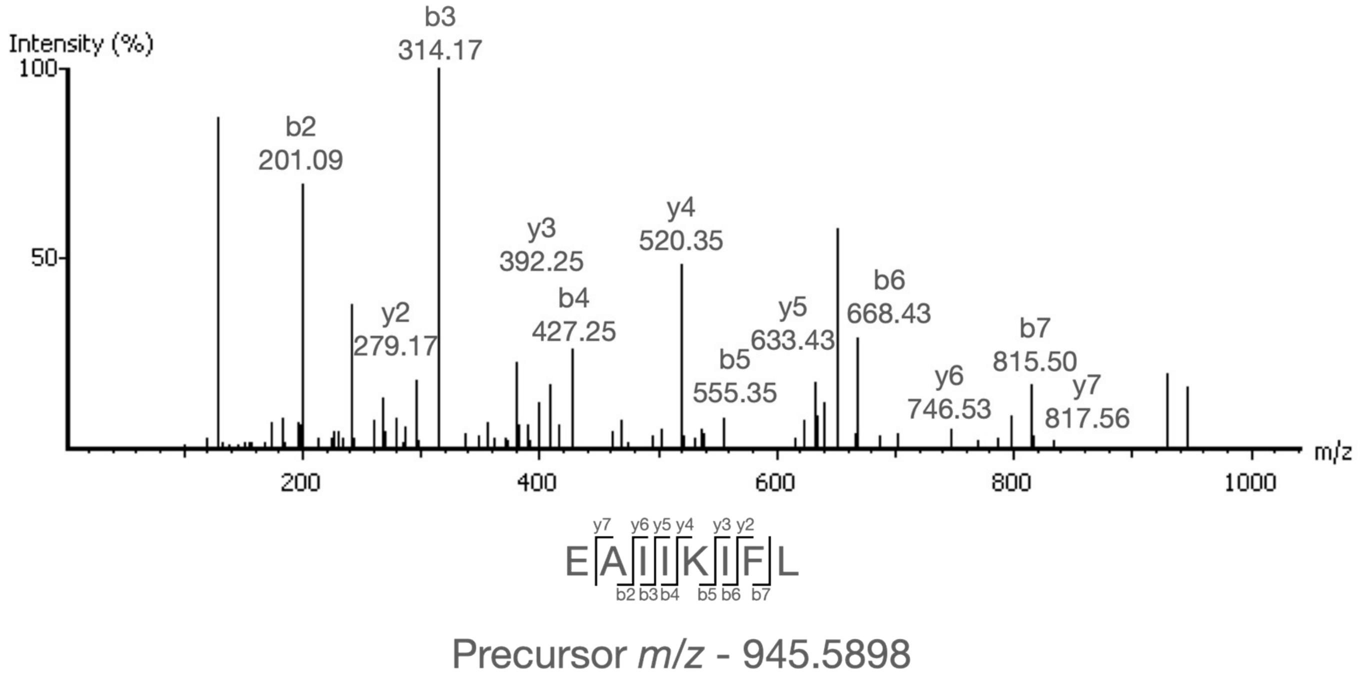

Fig. S11. MSMS analysis of fragment ions produced during assay of PEGA-Glu-surugamide A (the N-terminal Glu variant of surugamide A appended to the PEGA resin) and SurE. Only a linear peptide was produced. The peaks corresponding to each fragment ion are labelled with the representative *m/z* value. The nature of the precursor peptide is shown below the mass spectrum.

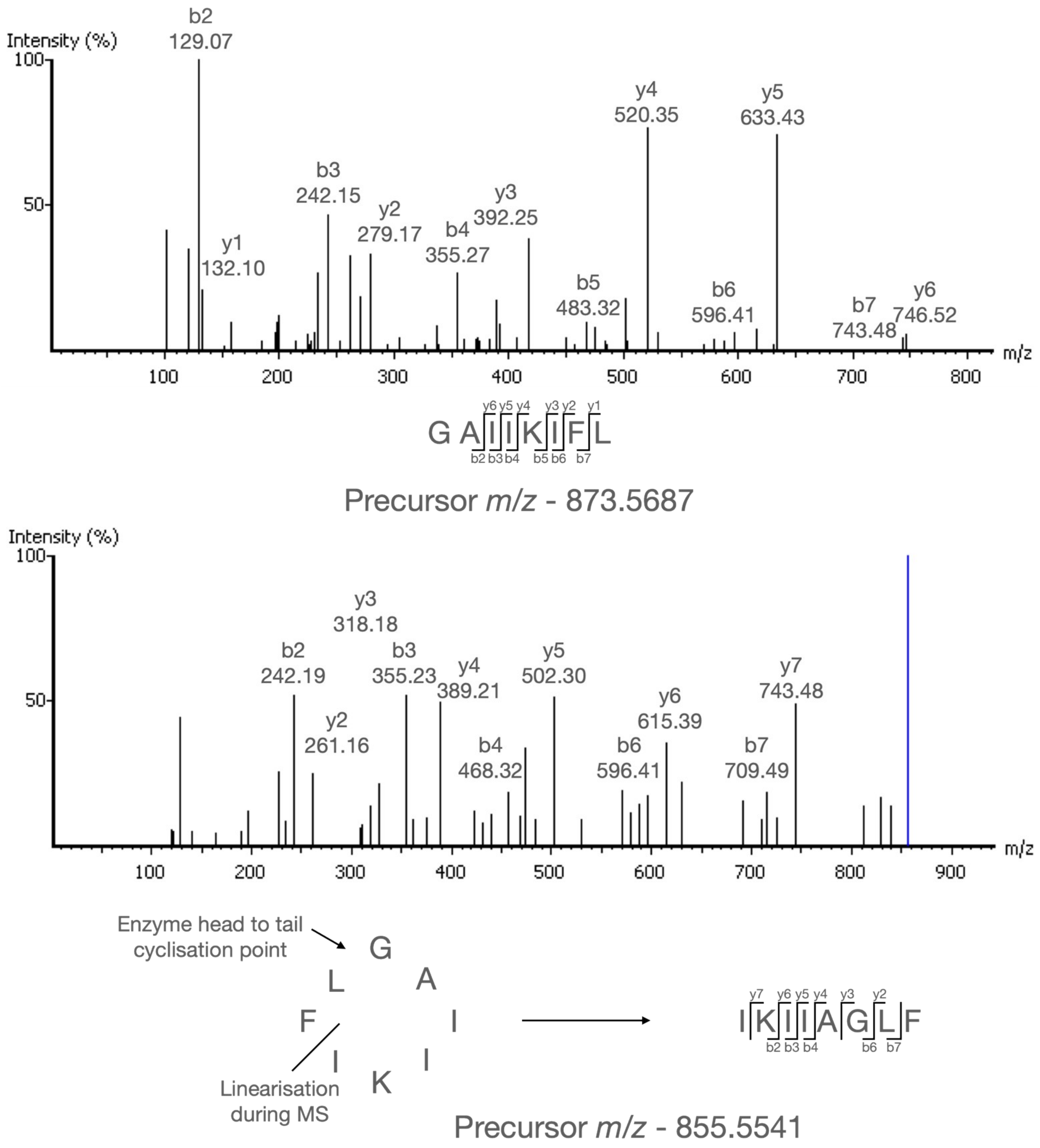

Fig. S12. MSMS analysis of fragment ions produced during assay of PEGA-Gly-surugamide A (the N-terminal Gly variant of surugamide A appended to the PEGA resin) and SurE. Both linear and cyclic peptides were produced. The peaks corresponding to each fragment ion are labelled with the representative *m/z* value. The nature of the precursor peptide is shown below the mass spectrum in each case.

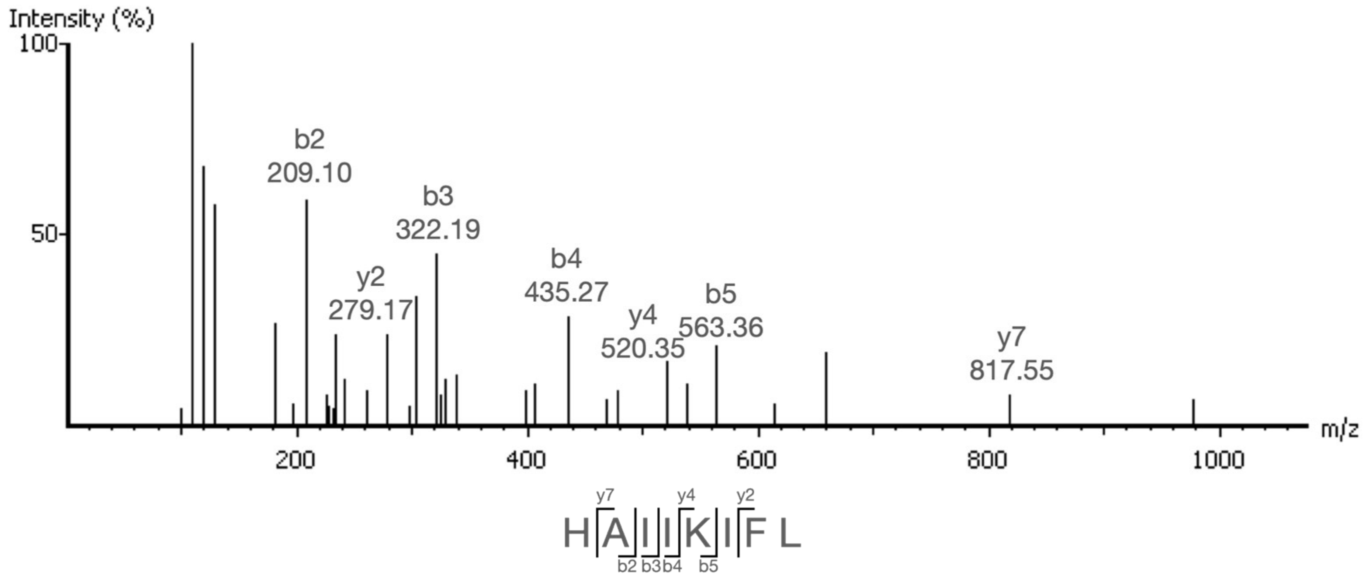

Precursor *m/z* - 953.6062

Fig. S13. MSMS analysis of fragment ions produced during assay of PEGA-His-surugamide A (the N-terminal His variant of surugamide A appended to the PEGA resin) and SurE. Only a linear peptide was produced. The peaks corresponding to each fragment ion are labelled with the representative *m/z* value. The nature of the precursor peptide is shown below the mass spectrum.

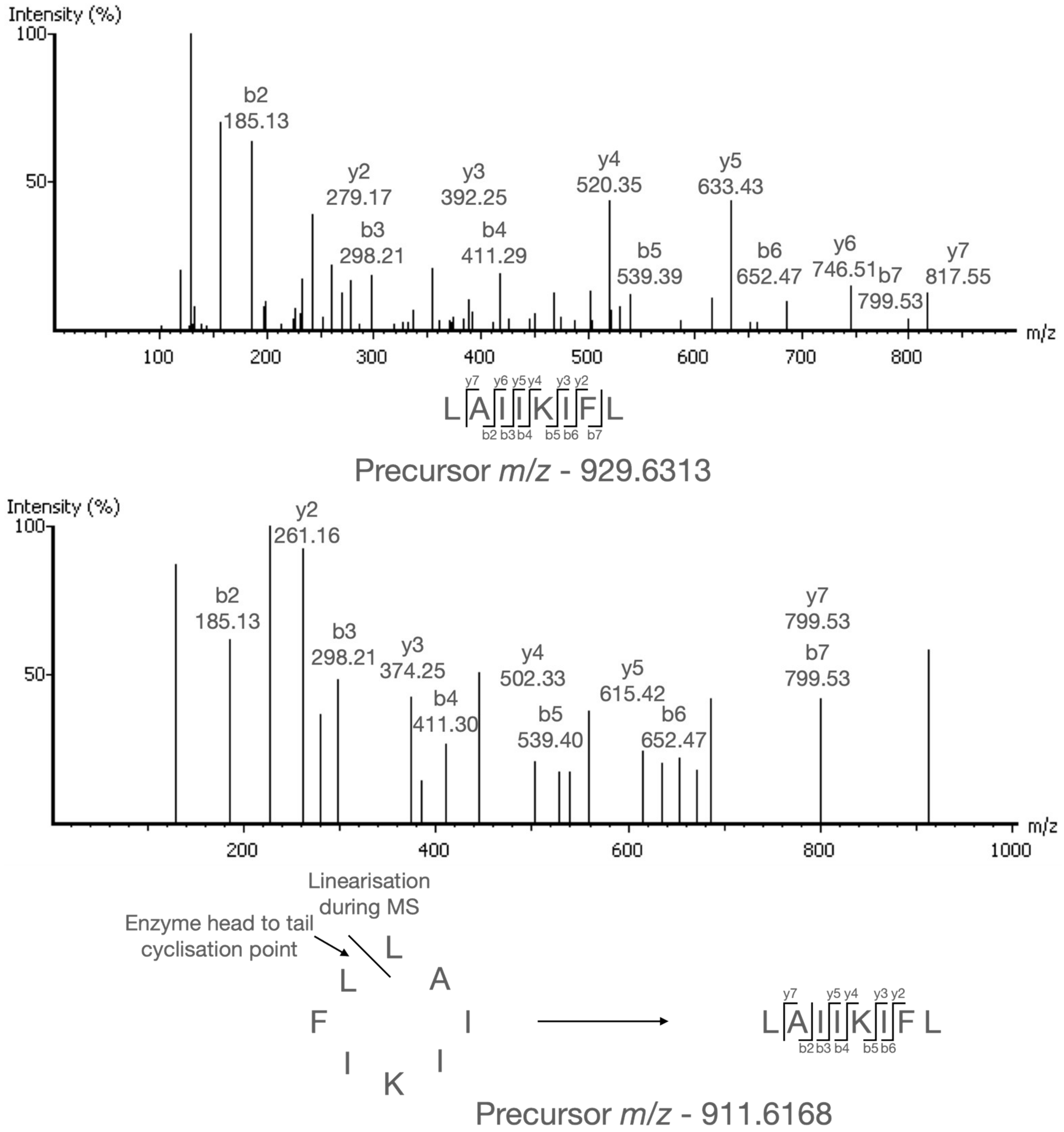

Fig. S14. MSMS analysis of fragment ions produced during assay of PEGA-Leu-surugamide A (the N-terminal Leu variant of surugamide A appended to the PEGA resin) and SurE. Both linear and cyclic peptides were produced. The peaks corresponding to each fragment ion are labelled with the representative *m/z* value. The nature of the precursor peptide is shown below the mass spectrum in each case.

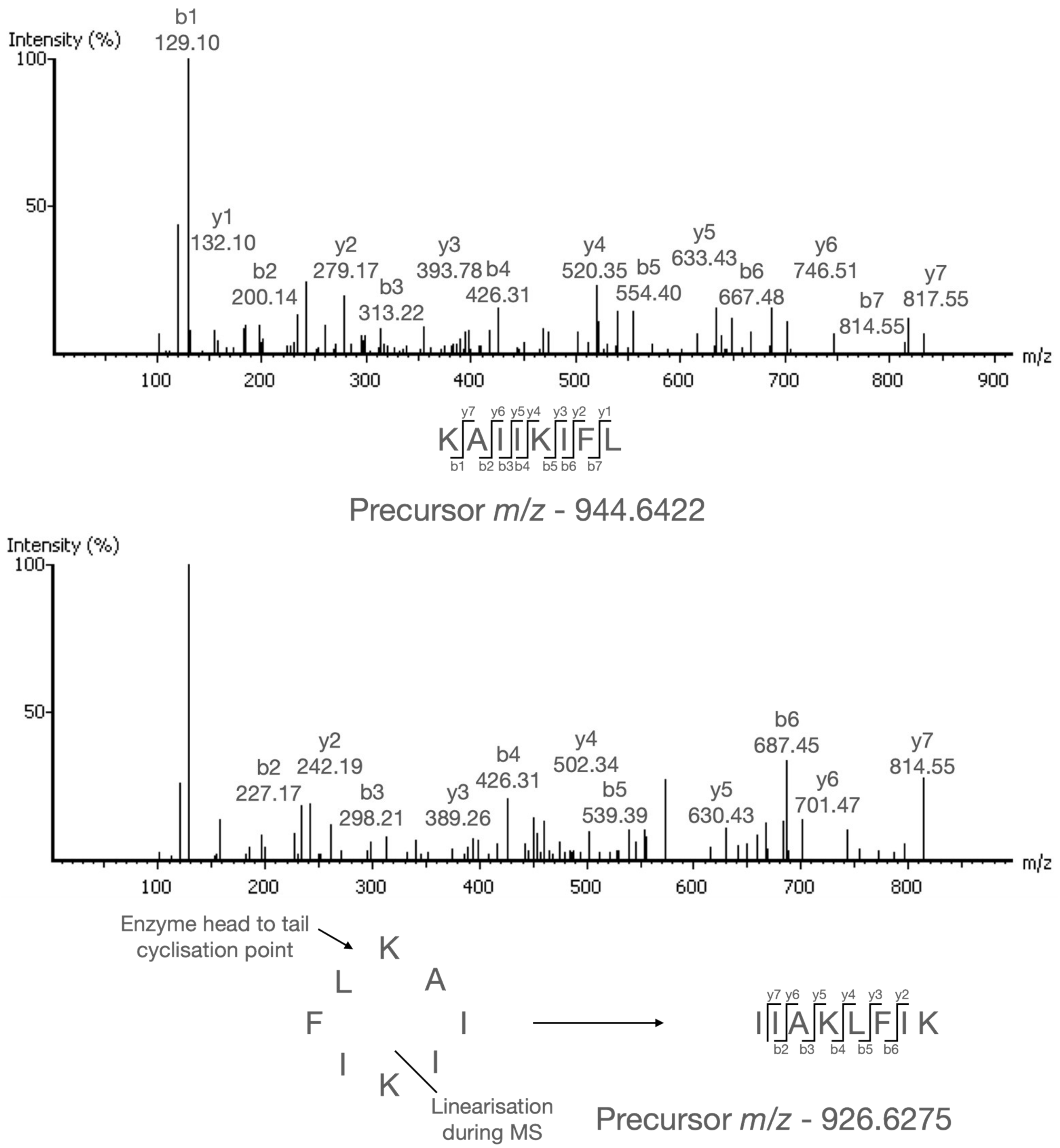

Fig. S15. MSMS analysis of fragment ions produced during assay of PEGA-Lys-surugamide A (the N-terminal Lys variant of surugamide A appended to the PEGA resin) and SurE. Both linear and cyclic peptides were produced. The peaks corresponding to each fragment ion are labelled with the representative *m/z* value. The nature of the precursor peptide is shown below the mass spectrum in each case.

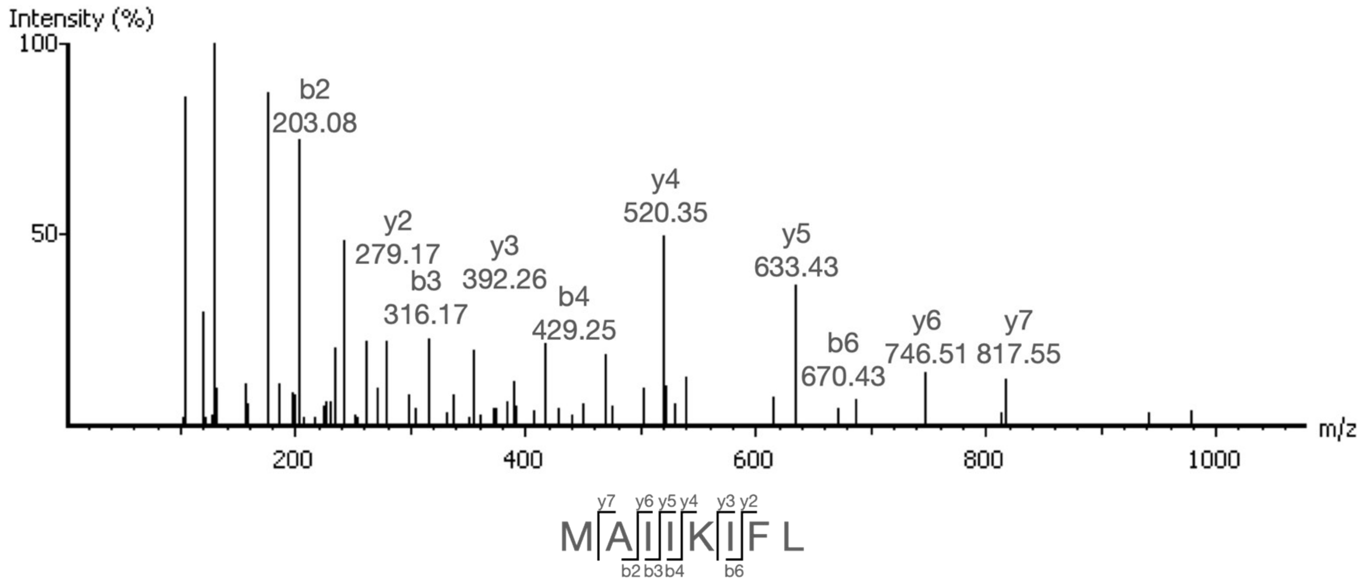

Precursor *m/z* - 947.5878

Fig. S16. MSMS analysis of fragment ions produced during assay of PEGA-Met-surugamide A (the N-terminal Met variant of surugamide A appended to the PEGA resin) and SurE. Only a linear peptide was produced. The peaks corresponding to each fragment ion are labelled with the representative *m/z* value. The nature of the precursor peptide is shown below the mass spectrum.

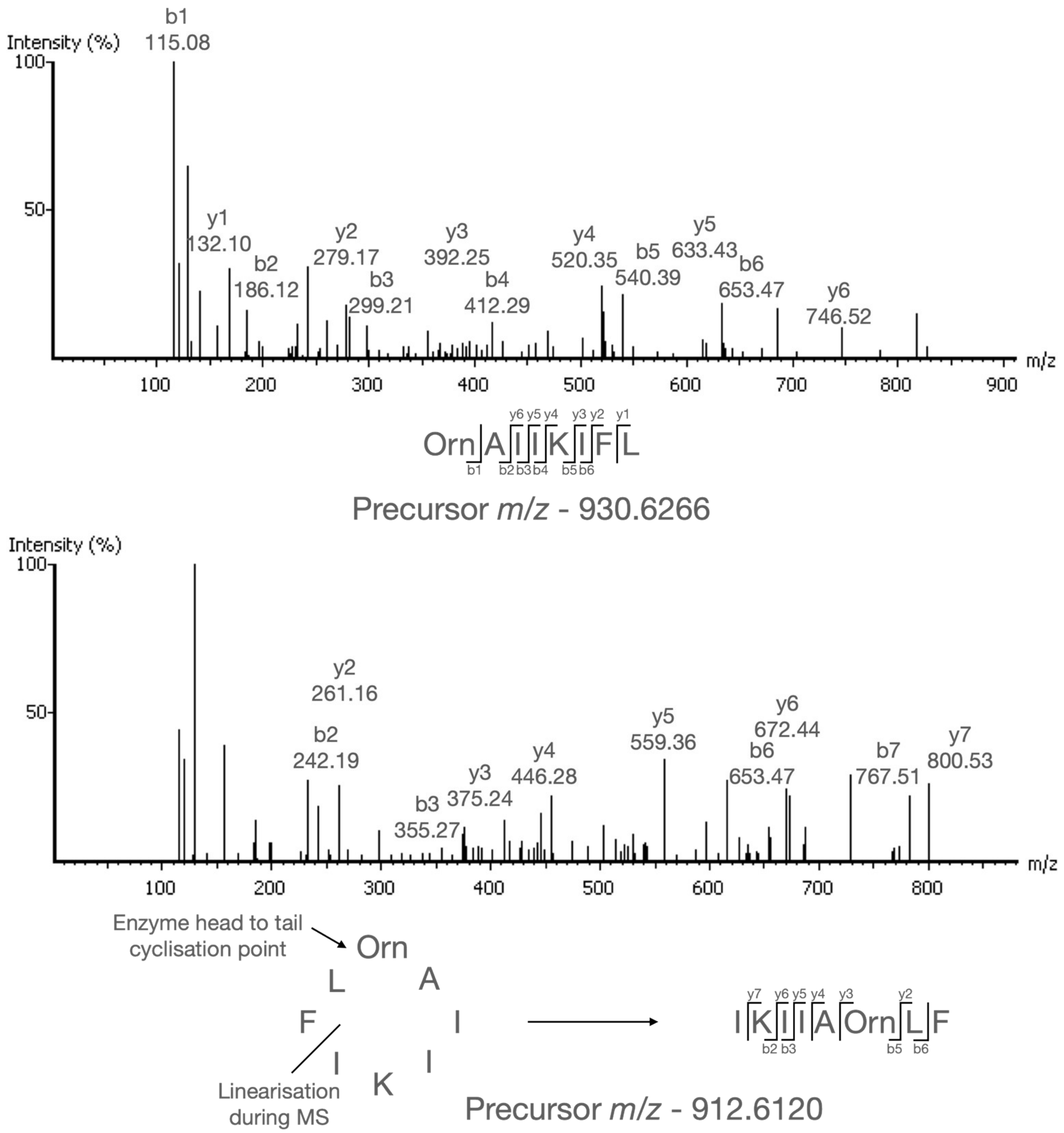

Fig. S17. MSMS analysis of fragment ions produced during assay of PEGA-Orn-surugamide A (the N-terminal Orn variant of surugamide A appended to the PEGA resin) and SurE. Both linear and cyclic peptides were produced. The peaks corresponding to each fragment ion are labelled with the representative *m/z* value. The nature of the precursor peptide is shown below the mass spectrum in each case.

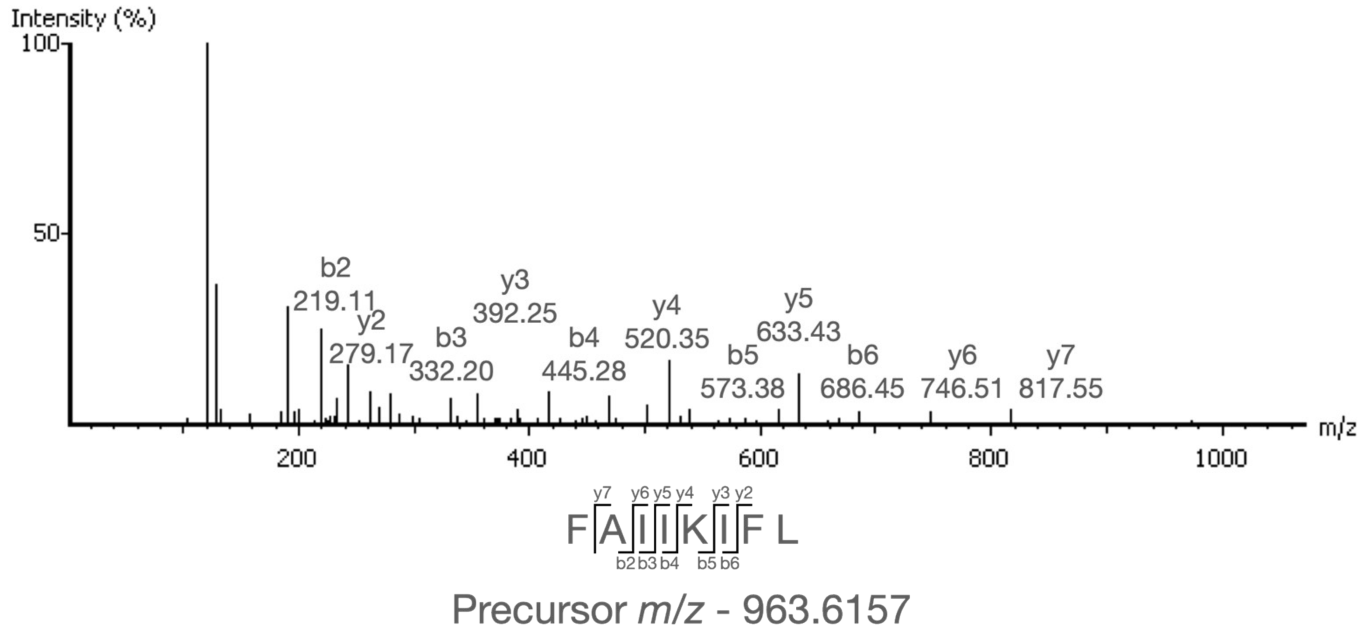

Fig. S18. MSMS analysis of fragment ions produced during assay of PEGA-Phe-surugamide A (the N-terminal Phe variant of surugamide A appended to the PEGA resin) and SurE. Only a linear peptide was produced. The peaks corresponding to each fragment ion are labelled with the representative *m/z* value. The nature of the precursor peptide is shown below the mass spectrum.

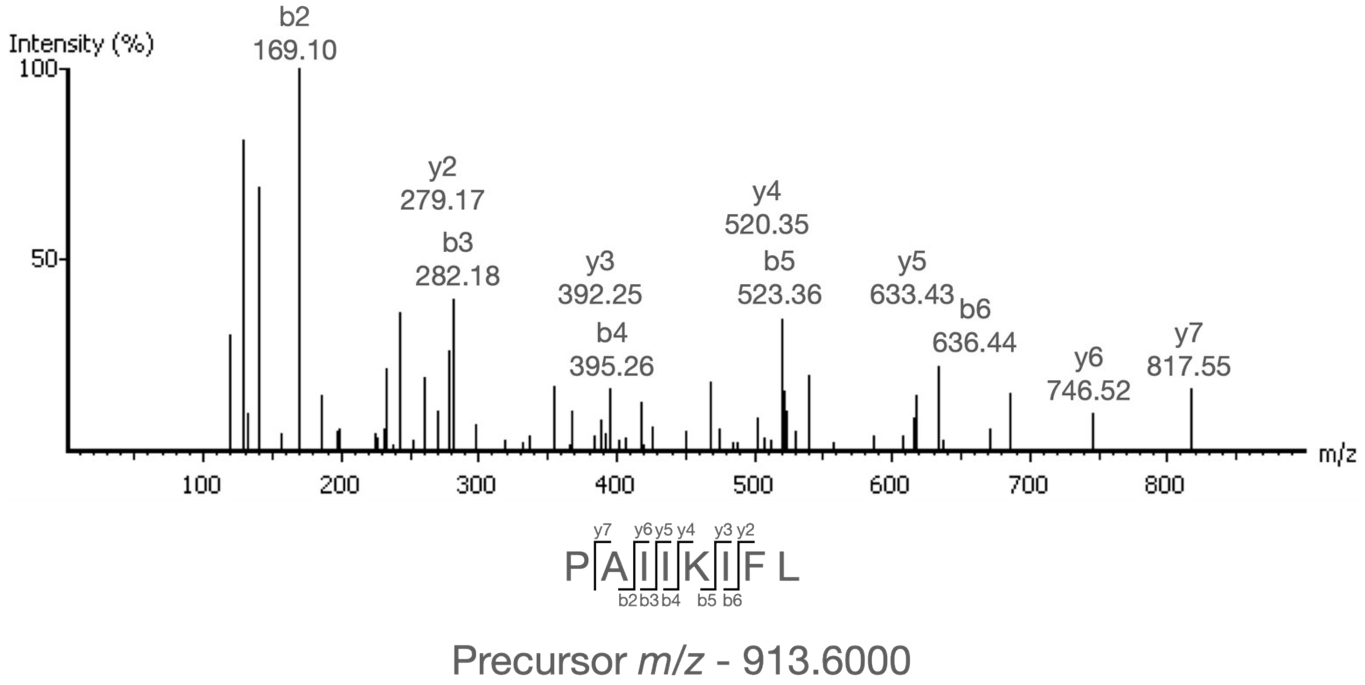

Fig. S19. MSMS analysis of fragment ions produced during assay of PEGA-Pro-surugamide A (the N-terminal Pro variant of surugamide A appended to the PEGA resin) and SurE. Only a linear peptide was produced. The peaks corresponding to each fragment ion are labelled with the representative *m/z* value. The nature of the precursor peptide is shown below the mass spectrum.

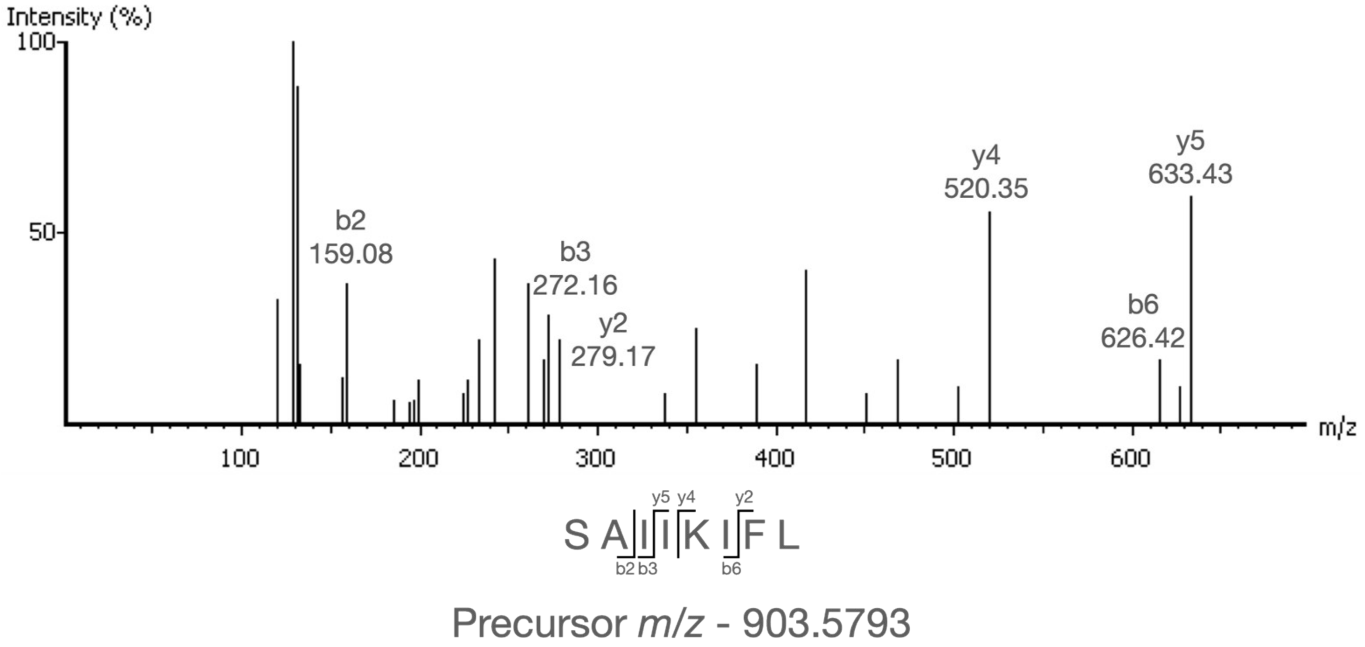

Fig. S20. MSMS analysis of fragment ions produced during assay of PEGA-Ser-surugamide A (the N-terminal Ser variant of surugamide A appended to the PEGA resin) and SurE. Only a linear peptide was produced. The peaks corresponding to each fragment ion are labelled with the representative *m/z* value. The nature of the precursor peptide is shown below the mass spectrum.

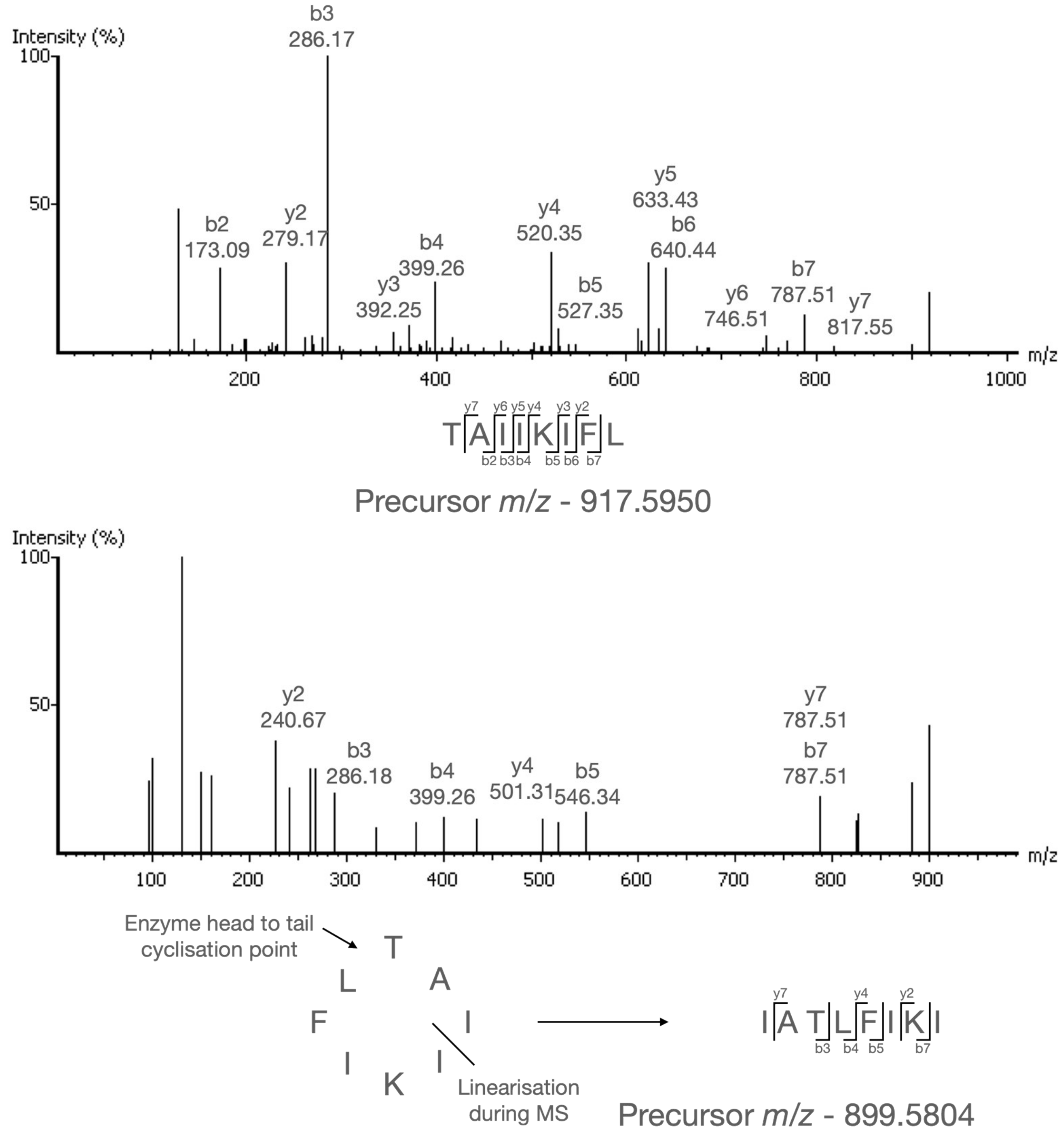

Fig. S21. MSMS analysis of fragment ions produced during assay of PEGA-Thr-surugamide A (the N-terminal Thr variant of surugamide A appended to the PEGA resin) and SurE. Both linear and cyclic peptides were produced. The peaks corresponding to each fragment ion are labelled with the representative *m/z* value. The nature of the precursor peptide is shown below the mass spectrum in each case.

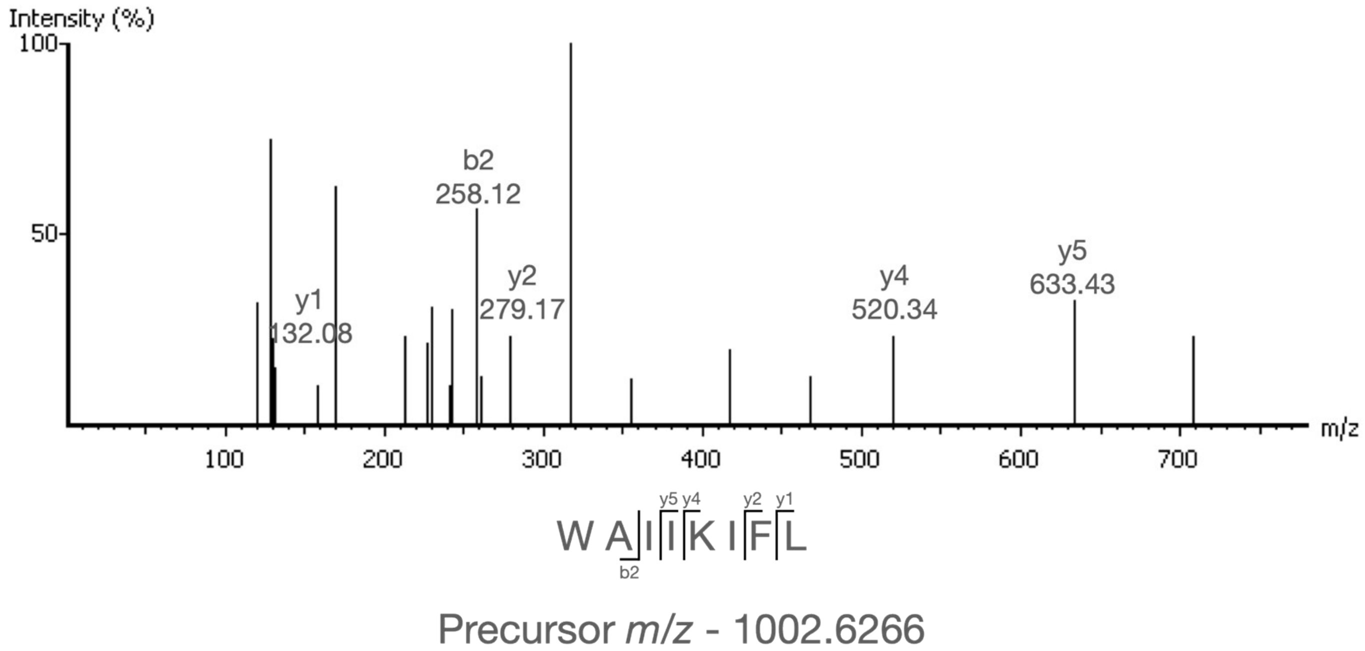

Fig. S22. MSMS analysis of fragment ions produced during assay of PEGA-Trp-surugamide A (the N-terminal Trp variant of surugamide A appended to the PEGA resin) and SurE. The peaks corresponding to each fragment ion are labelled with the representative *m/z* value. The nature of the precursor peptide is shown below the mass spectrum.

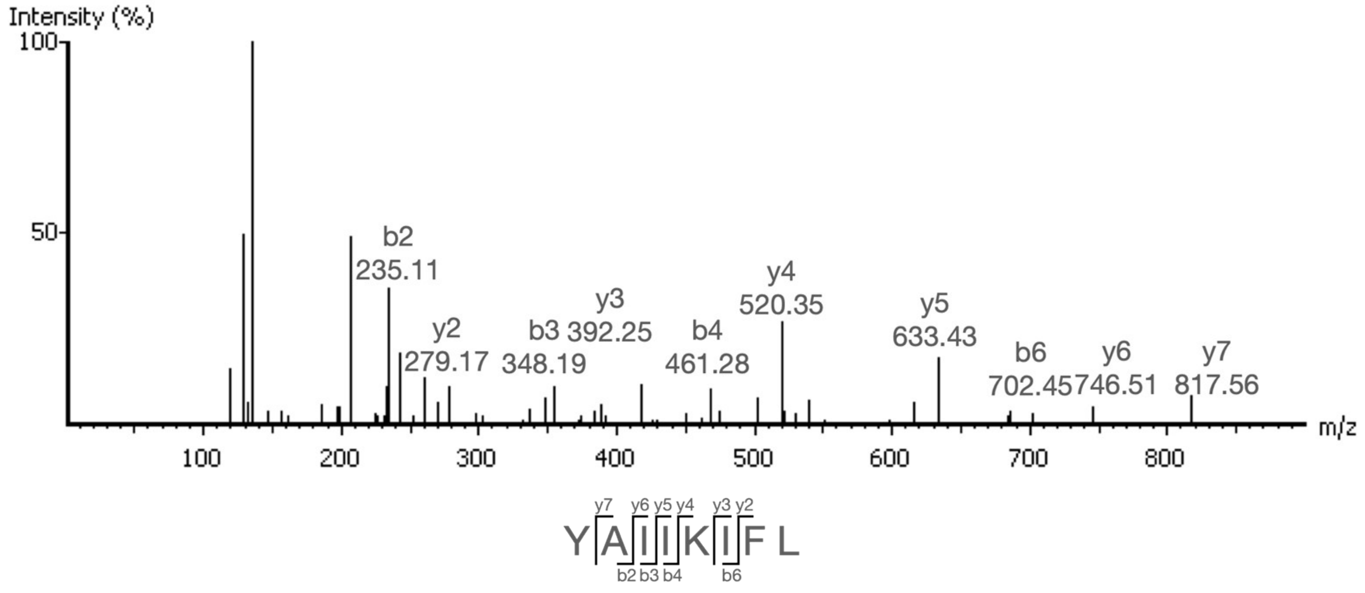

Fig. S23. MSMS analysis of fragment ions produced during assay of PEGA-Tyr-surugamide A (the N-terminal Tyr variant of surugamide A appended to the PEGA resin) and SurE. Only a linear peptide was produced. The peaks corresponding to each fragment ion are labelled with the representative *m/z* value. The nature of the precursor peptide is shown below the mass spectrum.

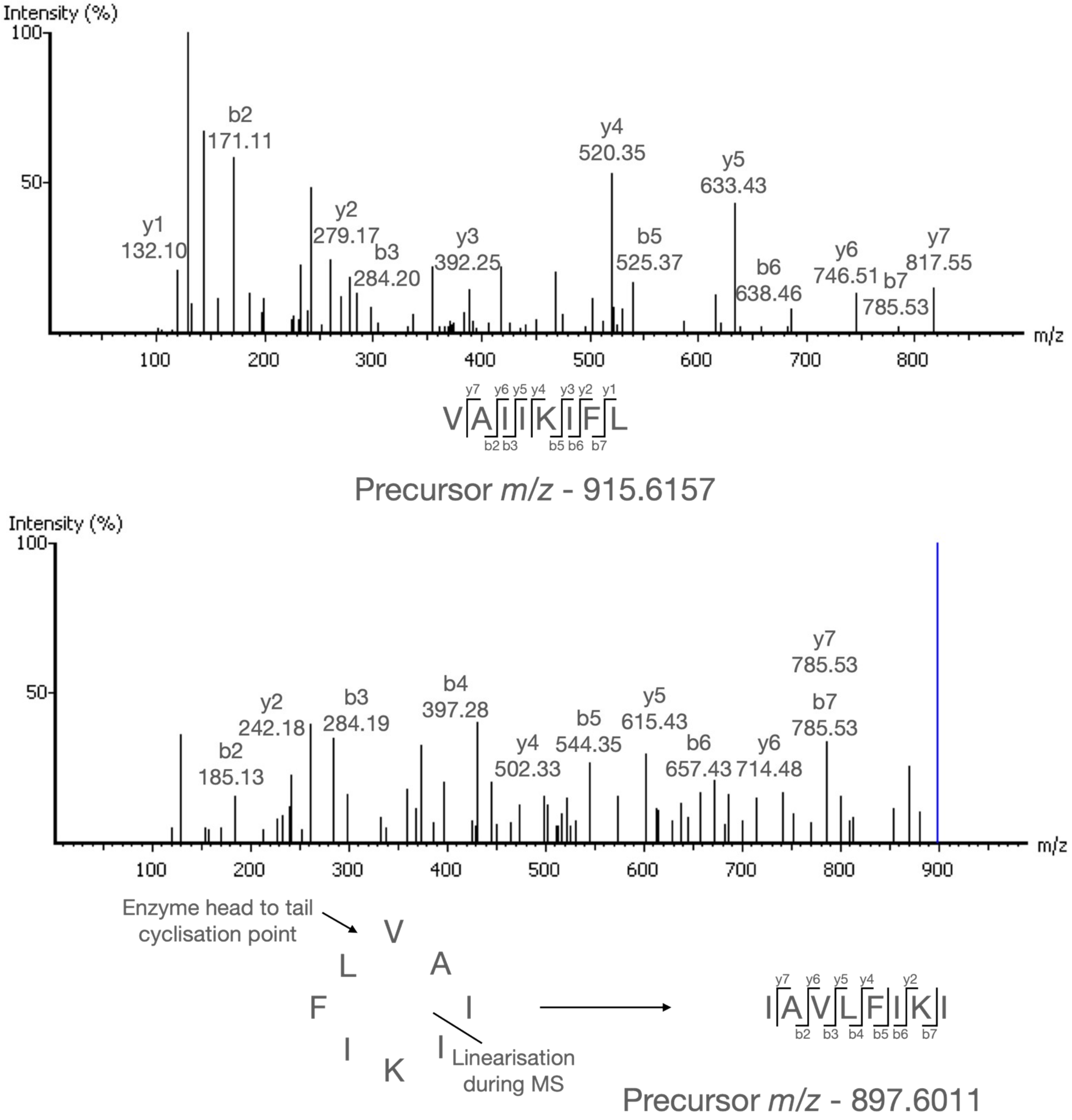

Fig. S24. MSMS analysis of fragment ions produced during assay of PEGA-Val-surugamide A (the N-terminal Val variant of surugamide A appended to the PEGA resin) and SurE. Both linear and cyclic peptides were produced. The peaks corresponding to each fragment ion are labelled with the representative *m/z* value. The nature of the precursor peptide is shown below the mass spectrum in each case.

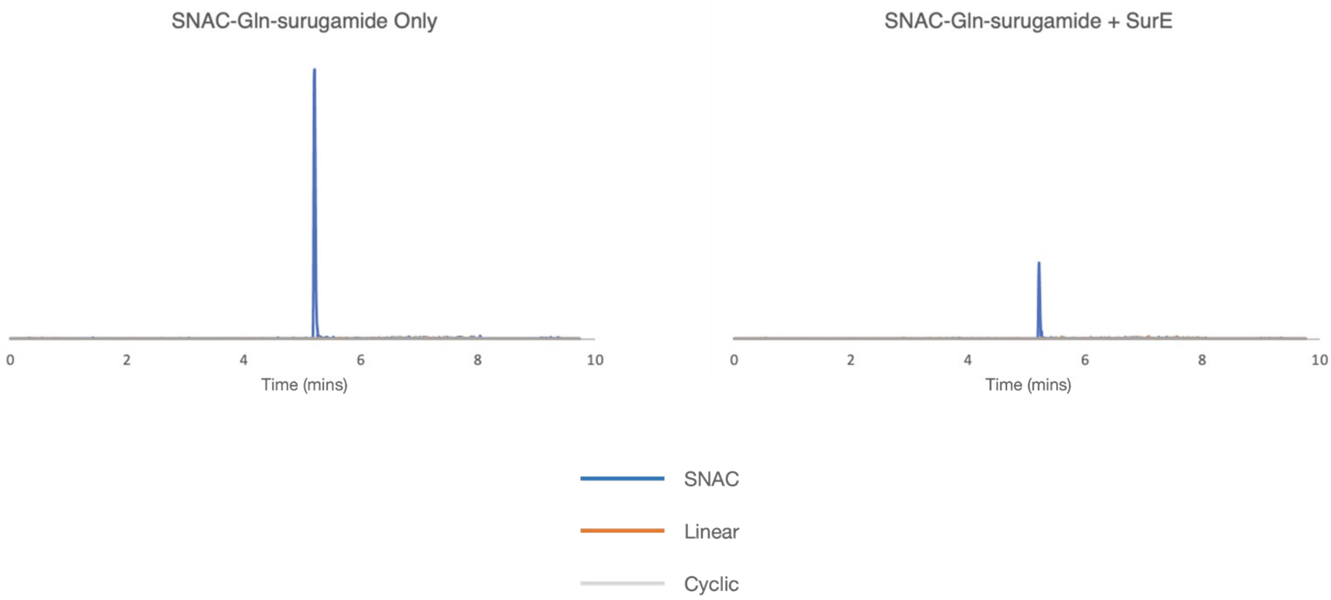

Fig. S25. LC-HRMS analysis of assays involving SNAC-Gln-surugamide A (the N-terminal Gln variant of SNAC-surugamide A) with (right) or without (left) SurE. Extracted ion chromatograms corresponding to the $[M+H]^+$ and $[M+Na]^+$ ions of the complete SNAC form of the peptide (blue), as well as the linear (orange) and cyclic (grey) forms are displayed. The *x* axis is displayed in units of time (mins) and the *y* axis was set to 8×10^4 for each of the chromatograms.

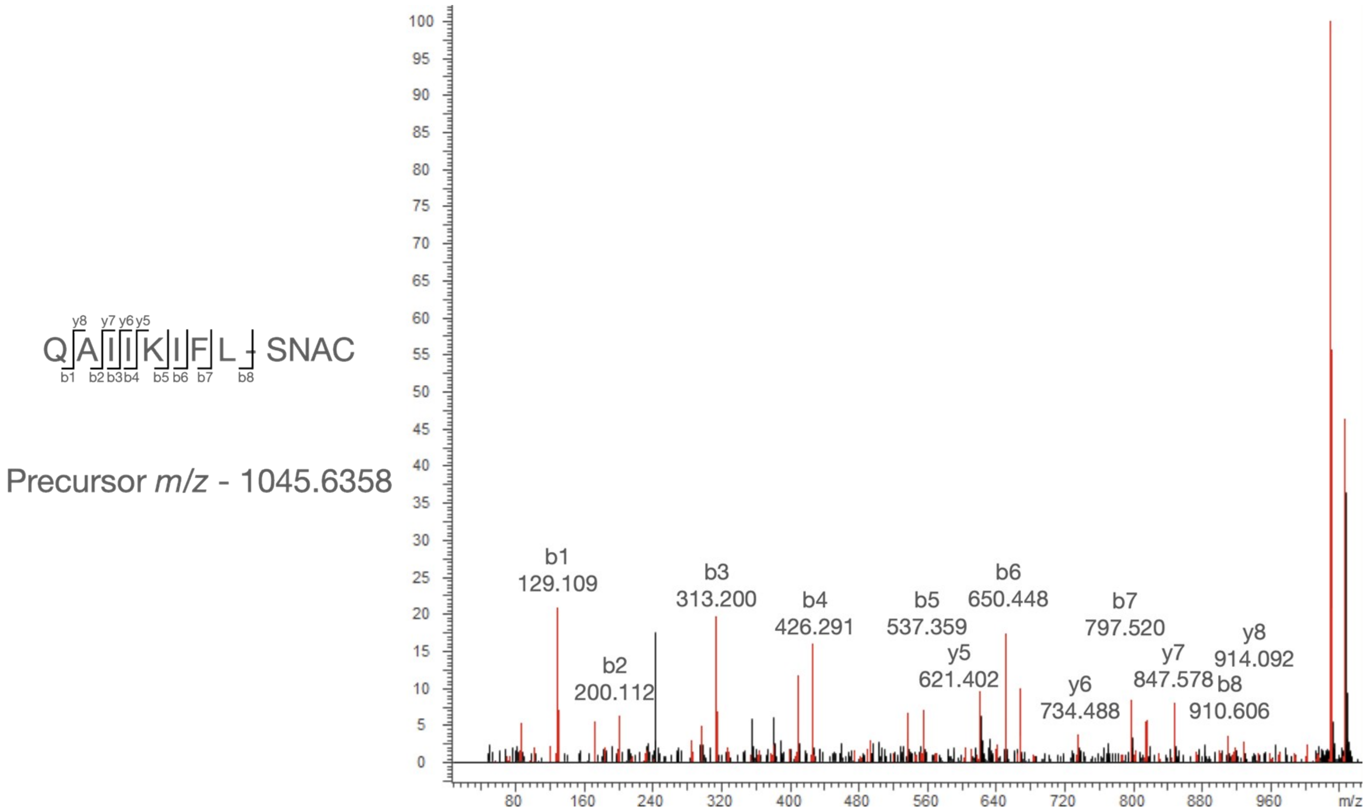

Fig. S26. MSMS analysis of fragment ions produced during assays of SNAC-Gln-surugamide A with SurE. The peaks corresponding to each fragment ion are labelled with the representative *m/z* value. The nature of the precursor peptide is shown beside the mass spectrum in each case.

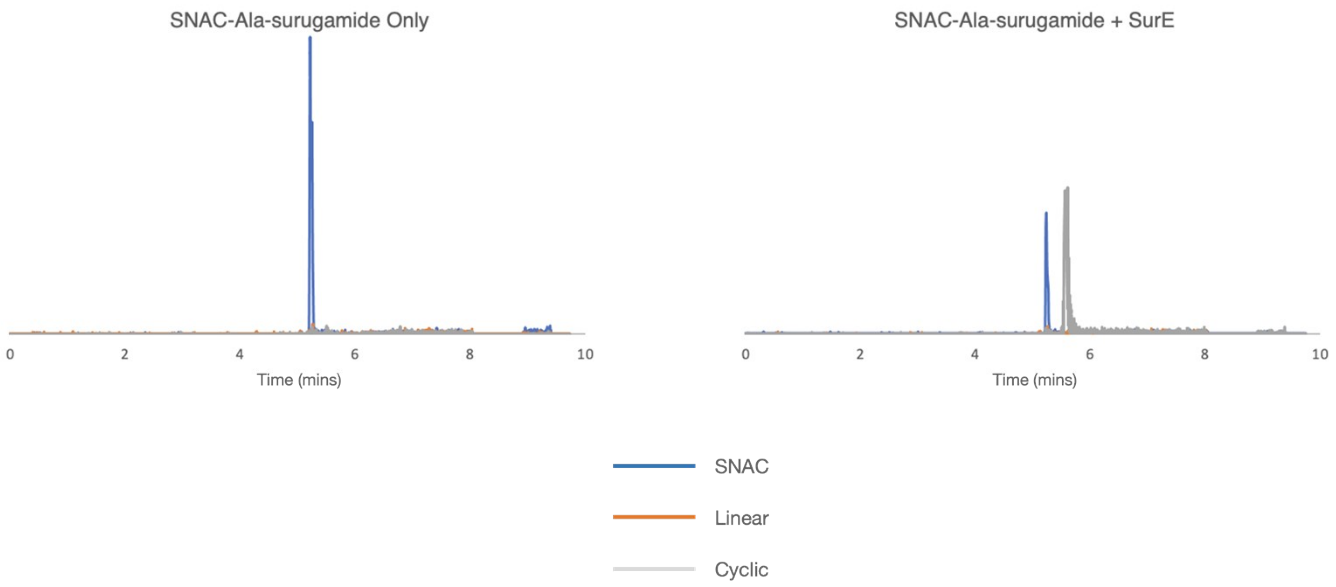

Fig. S27. LC-HRMS analysis of assays involving SNAC-Ala-surugamide A (the N-terminal Ala variant of SNAC-surugamide A) with (right) or without (left) SurE. Extracted ion chromatograms corresponding to the $[M+H]^+$ and $[M+Na]^+$ ions of the complete SNAC form of the peptide (blue), as well as the linear (orange) and cyclic (grey) forms are displayed. The *x* axis is displayed in units of time (mins) and the *y* axis was set to 4×10^4 for each of the chromatograms.

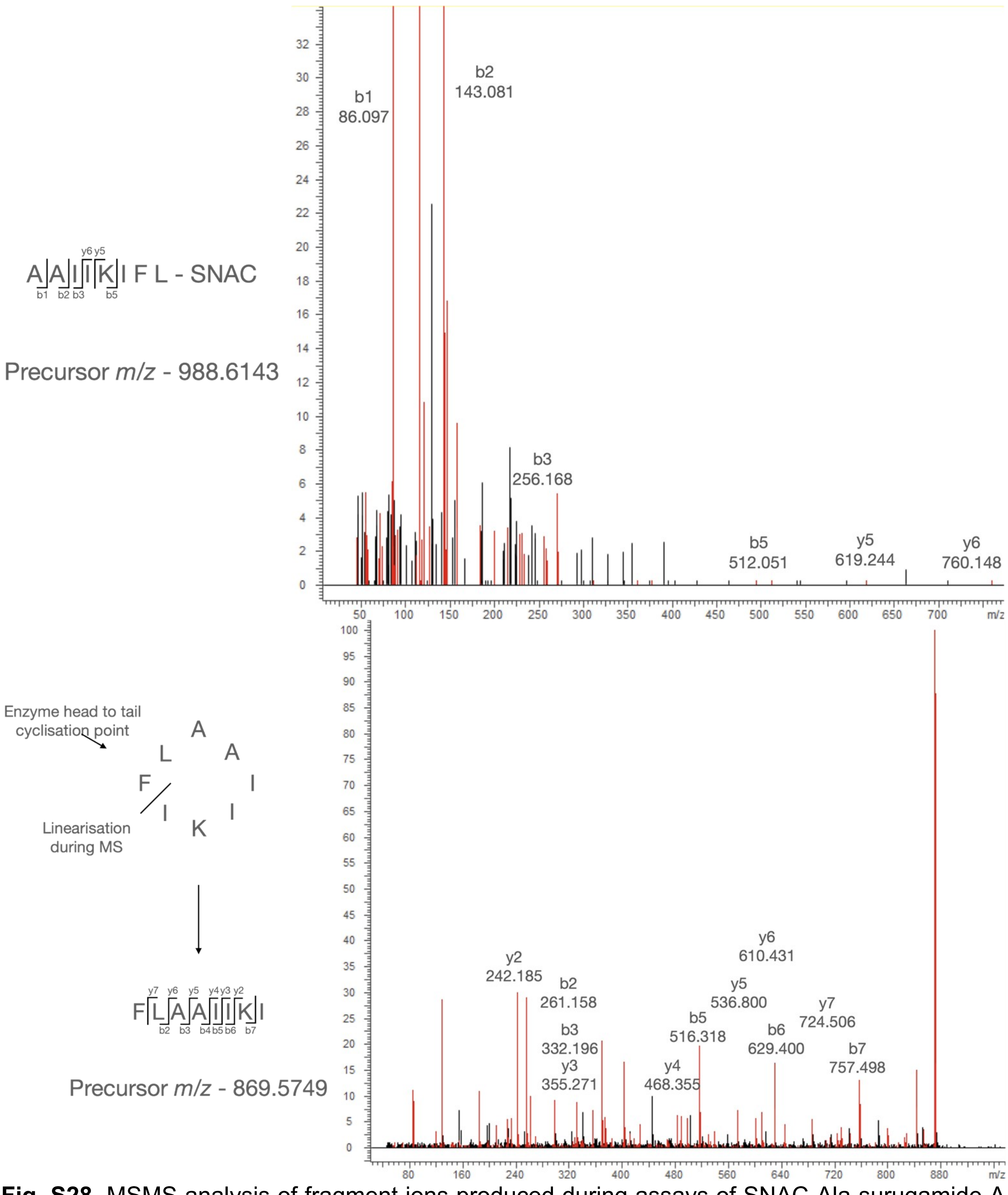

Fig. S28. MSMS analysis of fragment ions produced during assays of SNAC-Ala-surugamide A with SurE. The peaks corresponding to each fragment ion are labelled with the representative *m/z* value. The nature of the precursor peptide is shown beside the mass spectrum in each case.

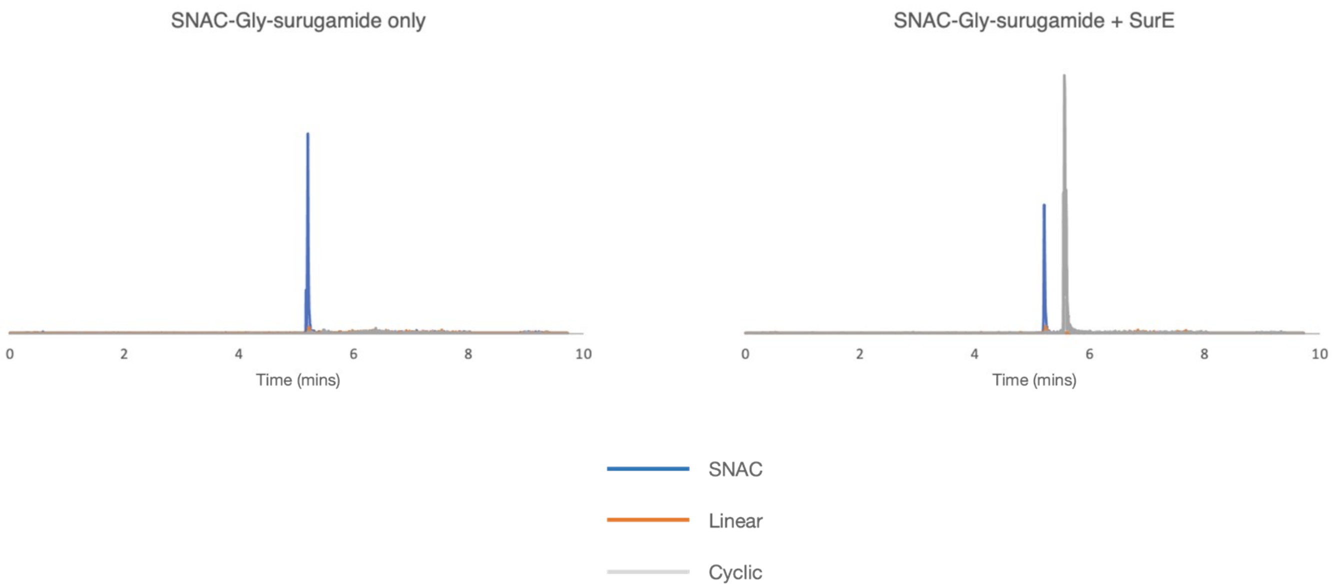

Fig. S29. LC-HRMS analysis of assays involving SNAC-Gly-surugamide A (the N-terminal Gly variant of SNAC-surugamide A) with (right) or without (left) SurE. Extracted ion chromatograms corresponding to the $[M+H]^+$ and $[M+Na]^+$ ions of the complete SNAC form of the peptide (blue), as well as the linear (orange) and cyclic (grey) forms are displayed. The *x* axis is displayed in units of time (mins) and the *y* axis was set to 1×10^5 for each of the chromatograms.

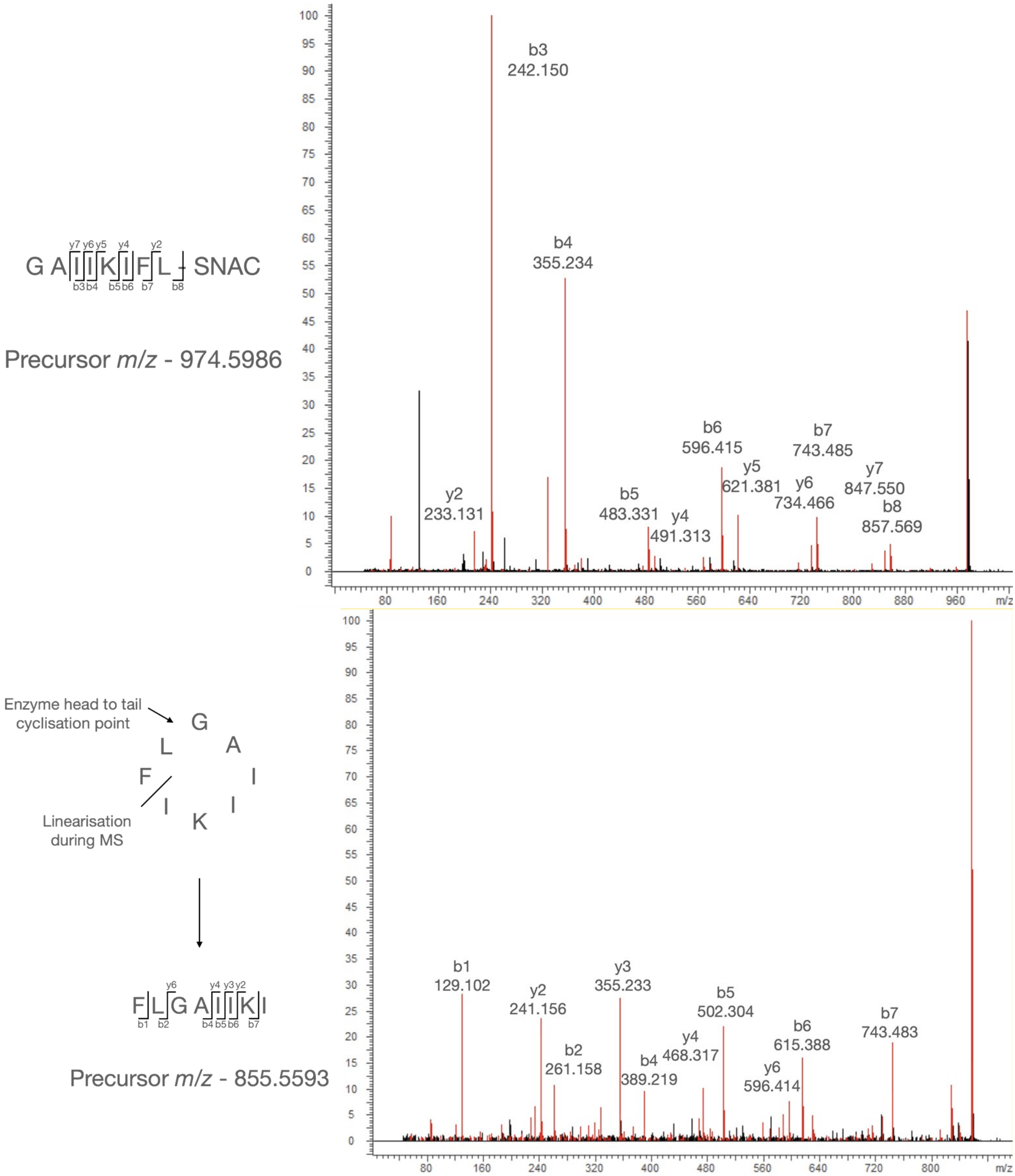

Fig. S30. MSMS analysis of fragment ions produced during assays of SNAC-Gly-surugamide A with SurE. The peaks corresponding to each fragment ion are labelled with the representative *m/z* value. The nature of the precursor peptide is shown beside the mass spectrum in each case.

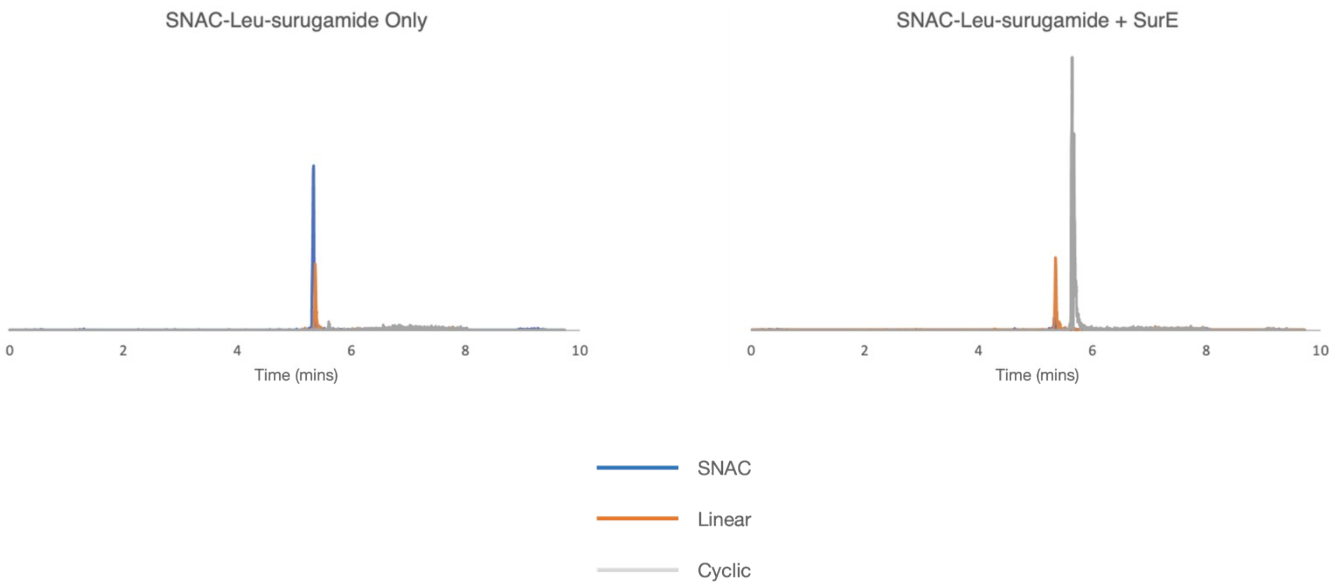

Fig. S31. LC-HRMS analysis of assays involving SNAC-Leu-surugamide A (the N-terminal Leu variant of SNAC-surugamide A) with (right) or without (left) SurE. Extracted ion chromatograms corresponding to the $[M+H]^+$ and $[M+Na]^+$ ions of the complete SNAC form of the peptide (blue), as well as the linear (orange) and cyclic (grey) forms are displayed. The *x* axis is displayed in units of time (mins) and the *y* axis was set to 8×10^4 for each of the chromatograms.

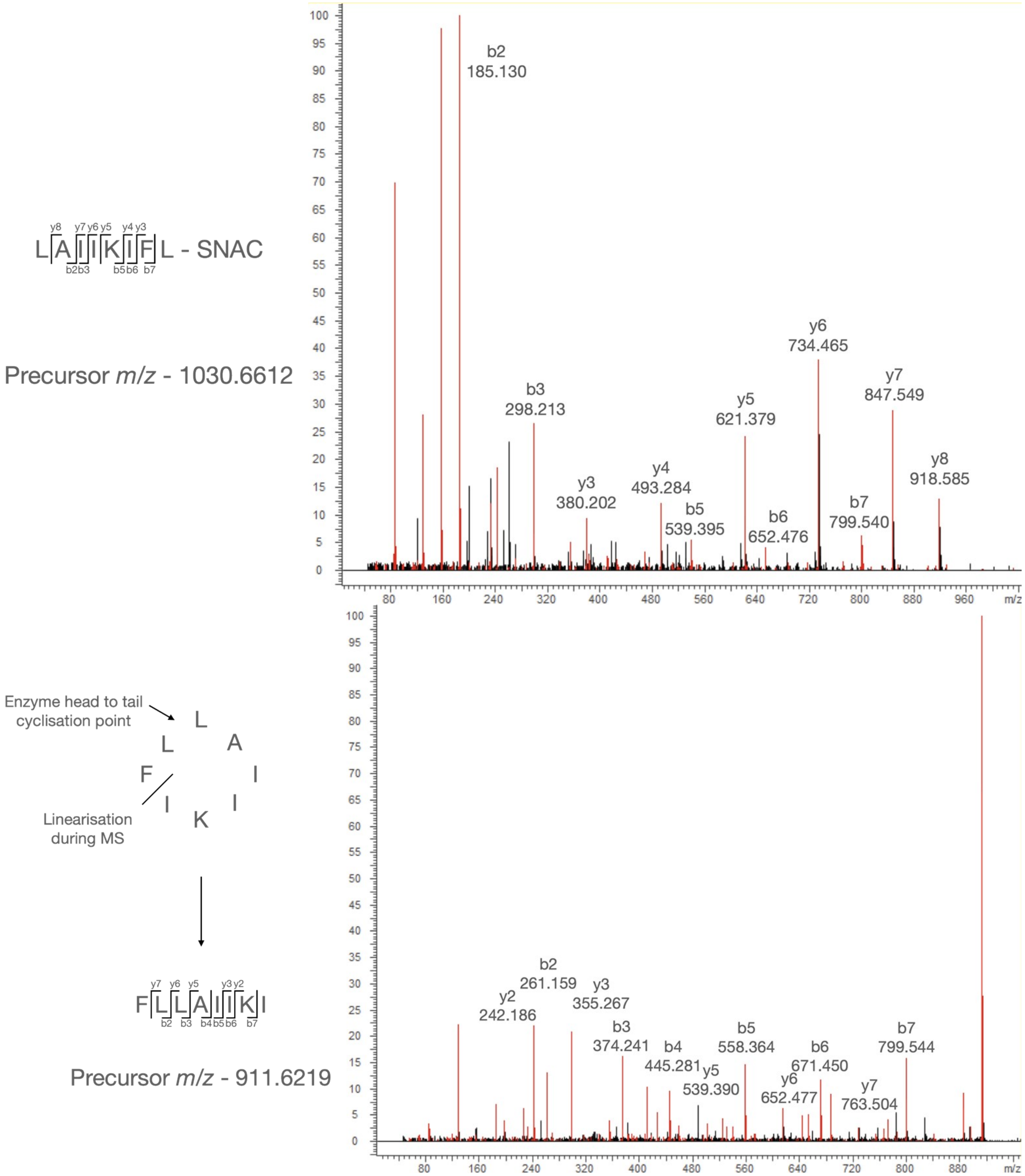

Fig. S32. MSMS analysis of fragment ions produced during assays of SNAC-Leu-surugamide A with SurE. The peaks corresponding to each fragment ion are labelled with the representative *m/z* value. The nature of the precursor peptide is shown beside the mass spectrum in each case.

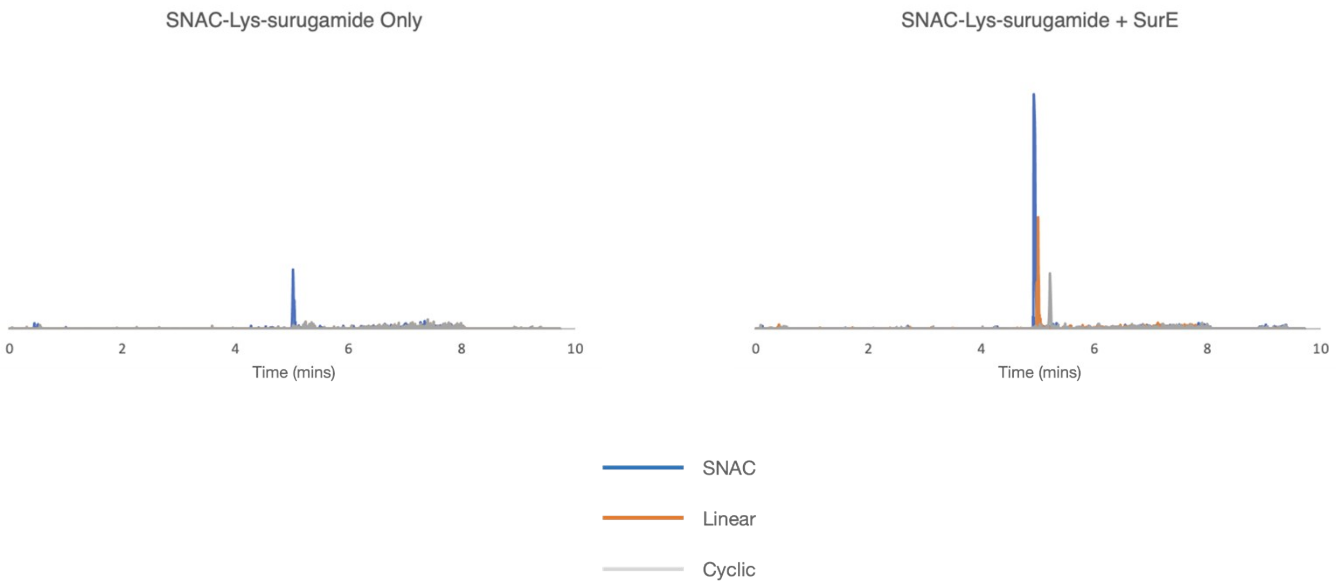

Fig. S33. LC-HRMS analysis of assays involving SNAC-Lys-surugamide A (the N-terminal Lys variant of SNAC-surugamide A) with (right) or without (left) SurE. Extracted ion chromatograms corresponding to the $[M+H]^+$ and $[M+Na]^+$ ions of the complete SNAC form of the peptide (blue), as well as the linear (orange) and cyclic (grey) forms are displayed. The *x* axis is displayed in units of time (mins) and the *y* axis was set to 3×10^4 for each of the chromatograms.

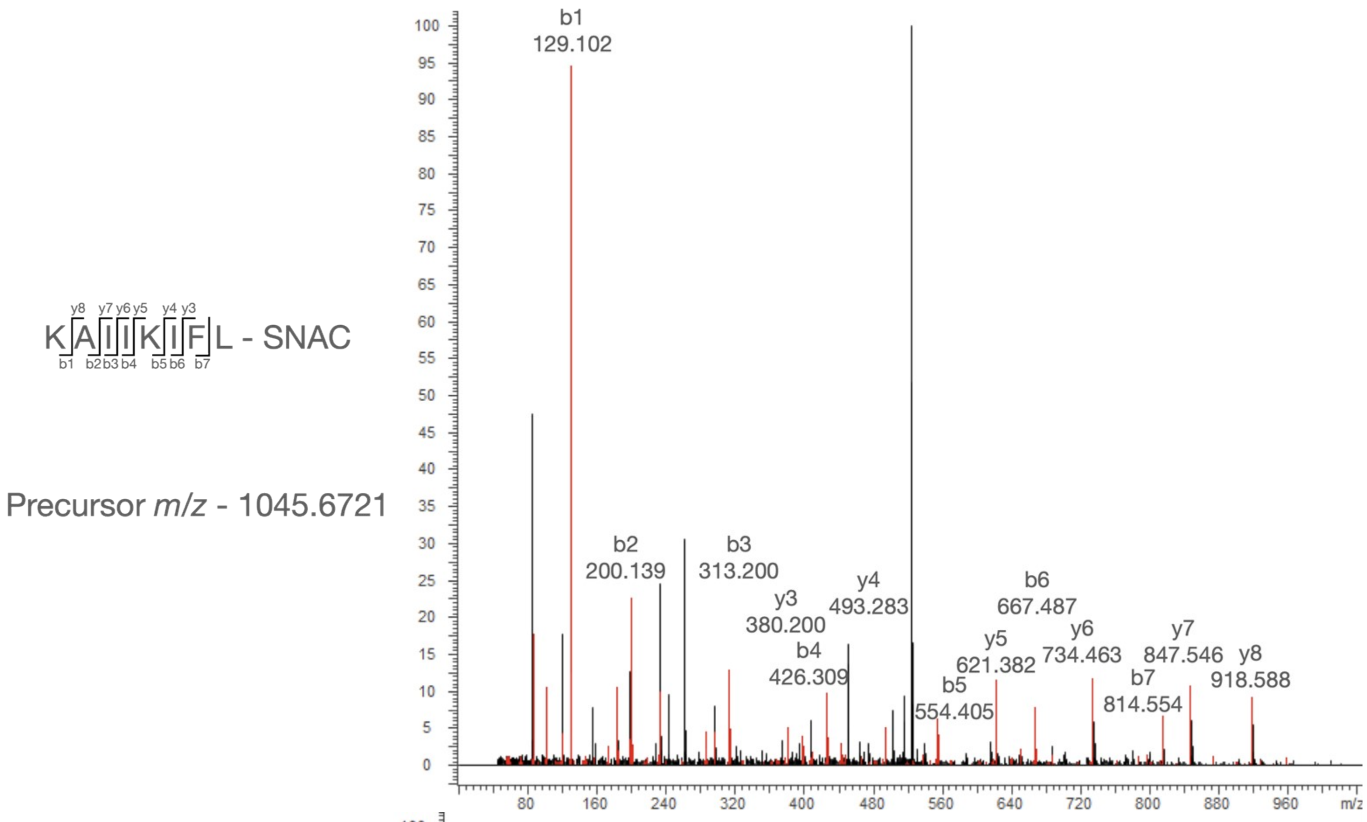

KAIIKIFL - SNAC
y8 y7 y6 y5 y4 y3
b1 b2 b3 b4 b5 b6 b7
Precursor m/z - 1045.6721
b1 129.102
b2 200.139
b3 313.200
y3 380.200
b4 426.309
y4 493.283
b5 554.405
y5 621.382
b6 667.487
y6 734.463
b7 814.554
y7 847.546
y8 918.588
m/z

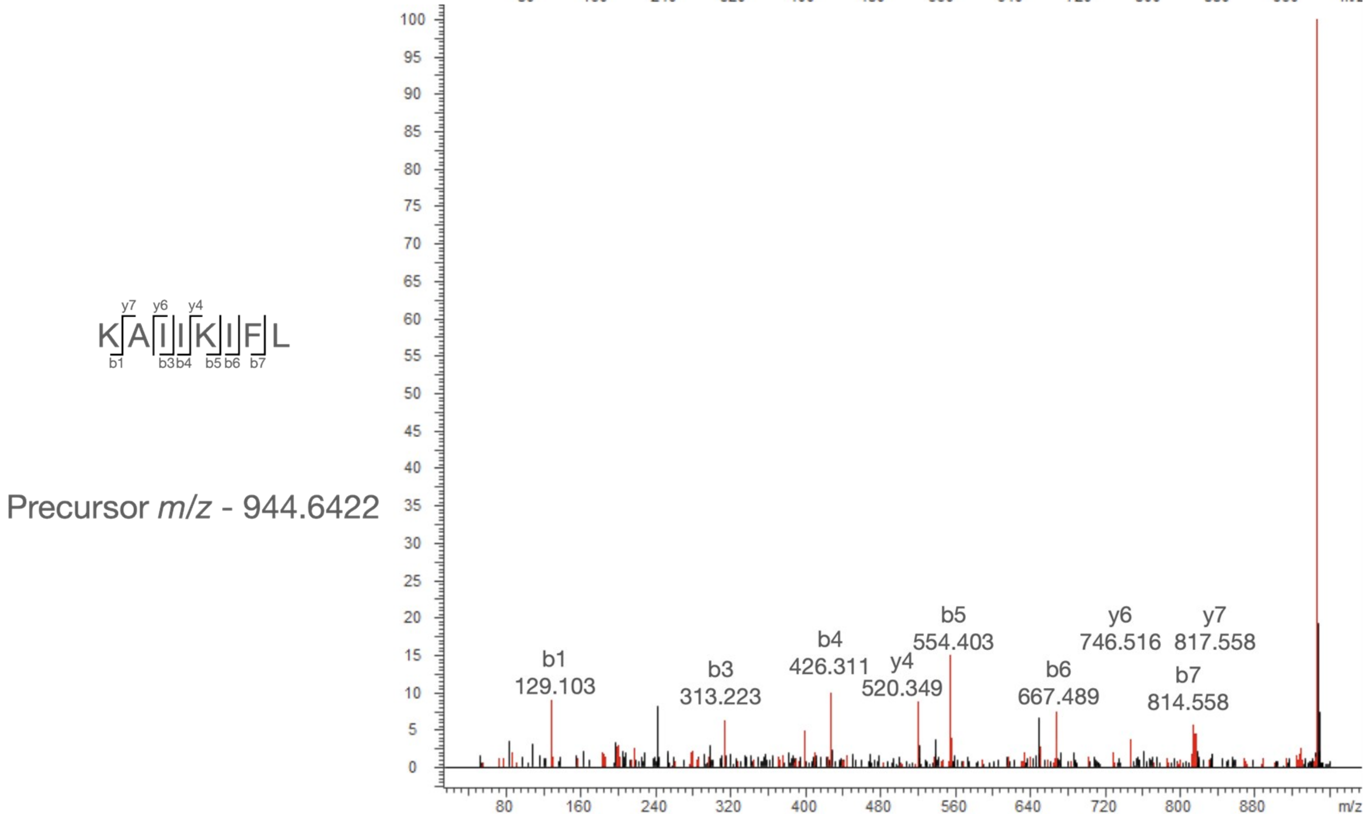

KAIIKIFL
y7 y6 y4
b1 b3 b4 b5 b6 b7
Precursor m/z - 944.6422
b1 129.103
b3 313.223
b4 426.311
y4 520.349
b5 554.403
b6 667.489
y6 746.516
y7 817.558
b7 814.558
m/z

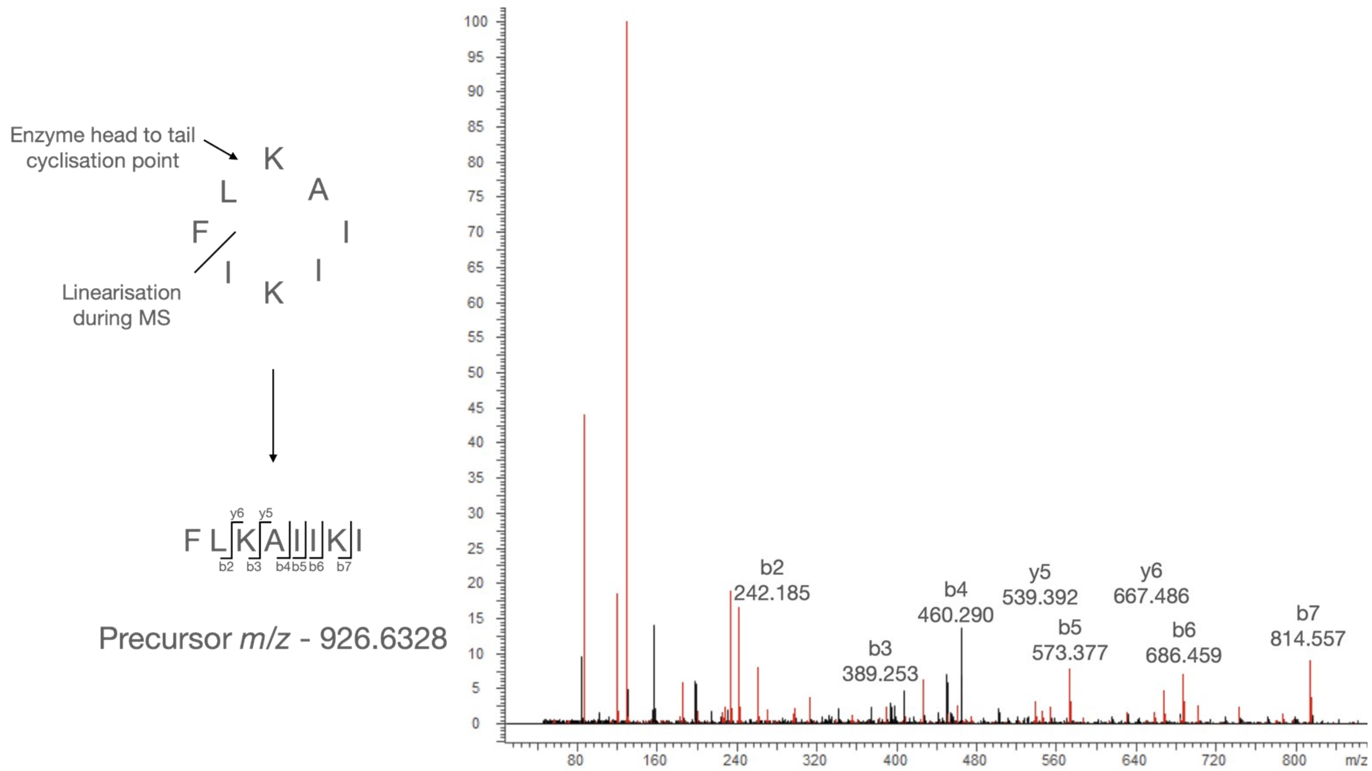

Fig. S34. MSMS analysis of fragment ions produced during assays of SNAC-Lys-surugamide A with SurE. The peaks corresponding to each fragment ion are labelled with the representative *m/z* value. The nature of the precursor peptide is shown beside the mass spectrum in each case.

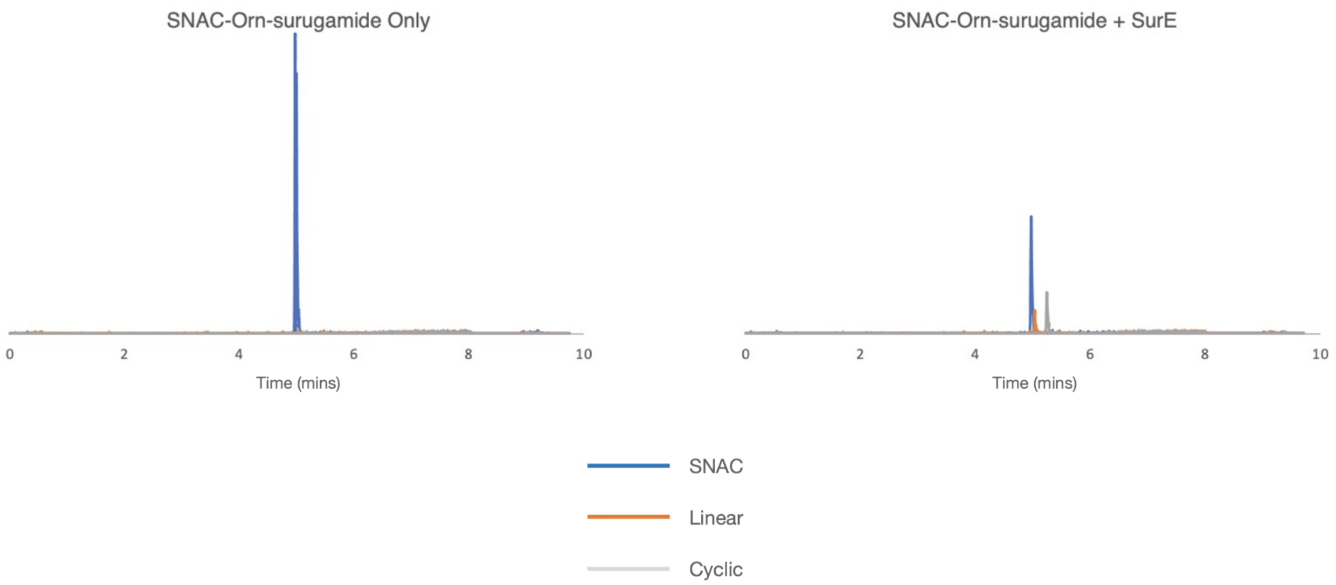

Fig. S35. LC-HRMS analysis of assays involving SNAC-Orn-surugamide A (the N-terminal Orn variant of SNAC-surugamide A) with (right) or without (left) SurE. Extracted ion chromatograms corresponding to the $[M+H]^+$ and $[M+Na]^+$ ions of the complete SNAC form of the peptide (blue), as well as the linear (orange) and cyclic (grey) forms are displayed. The *x* axis is displayed in units of time (mins) and the *y* axis was set to 5×10^4 for each of the chromatograms.

Orn A I I K I F L - SNAC

Precursor m/z - 1031.6565

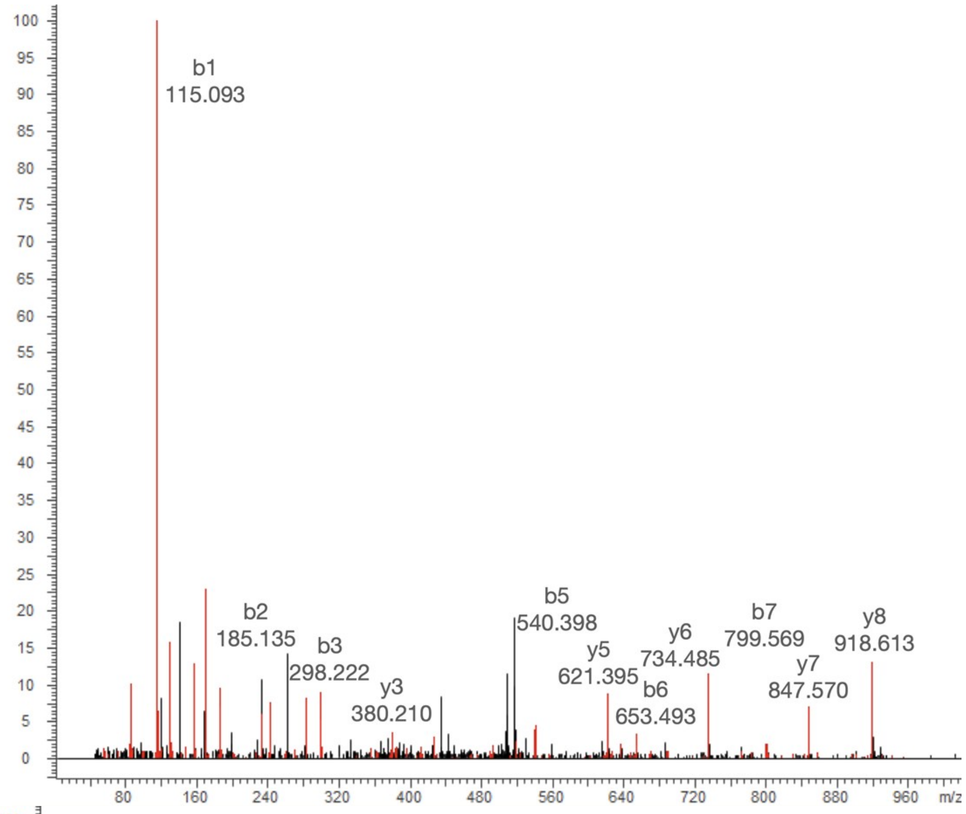

Orn A I I K I F L

Precursor m/z - 930.6266

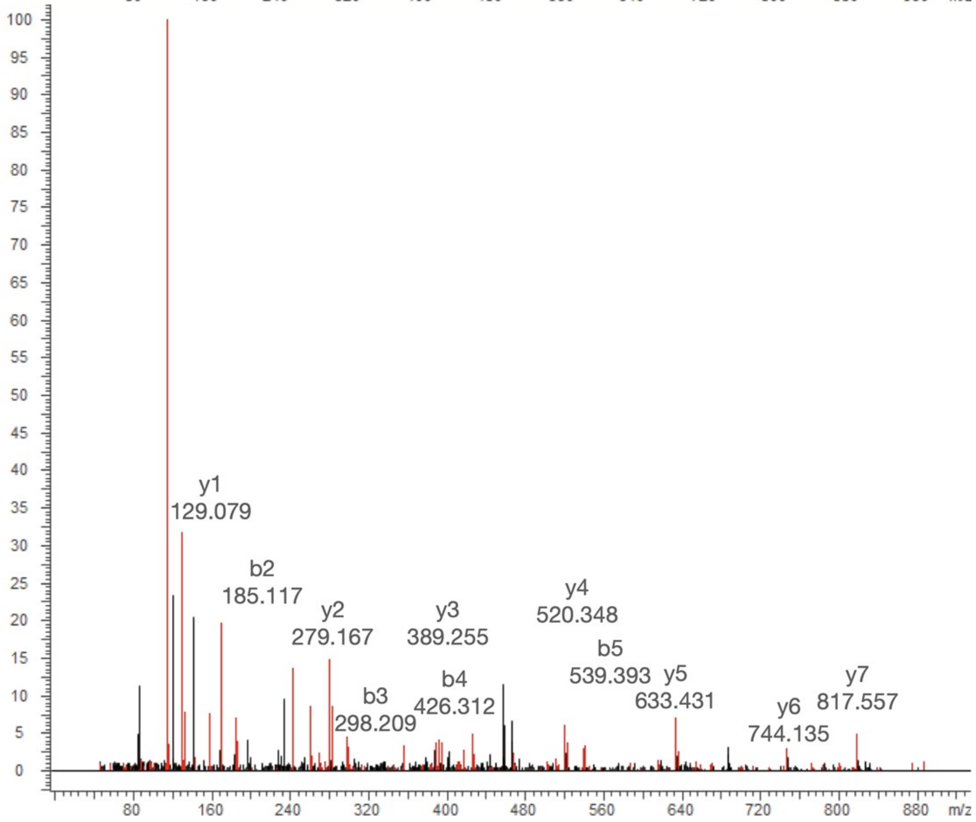

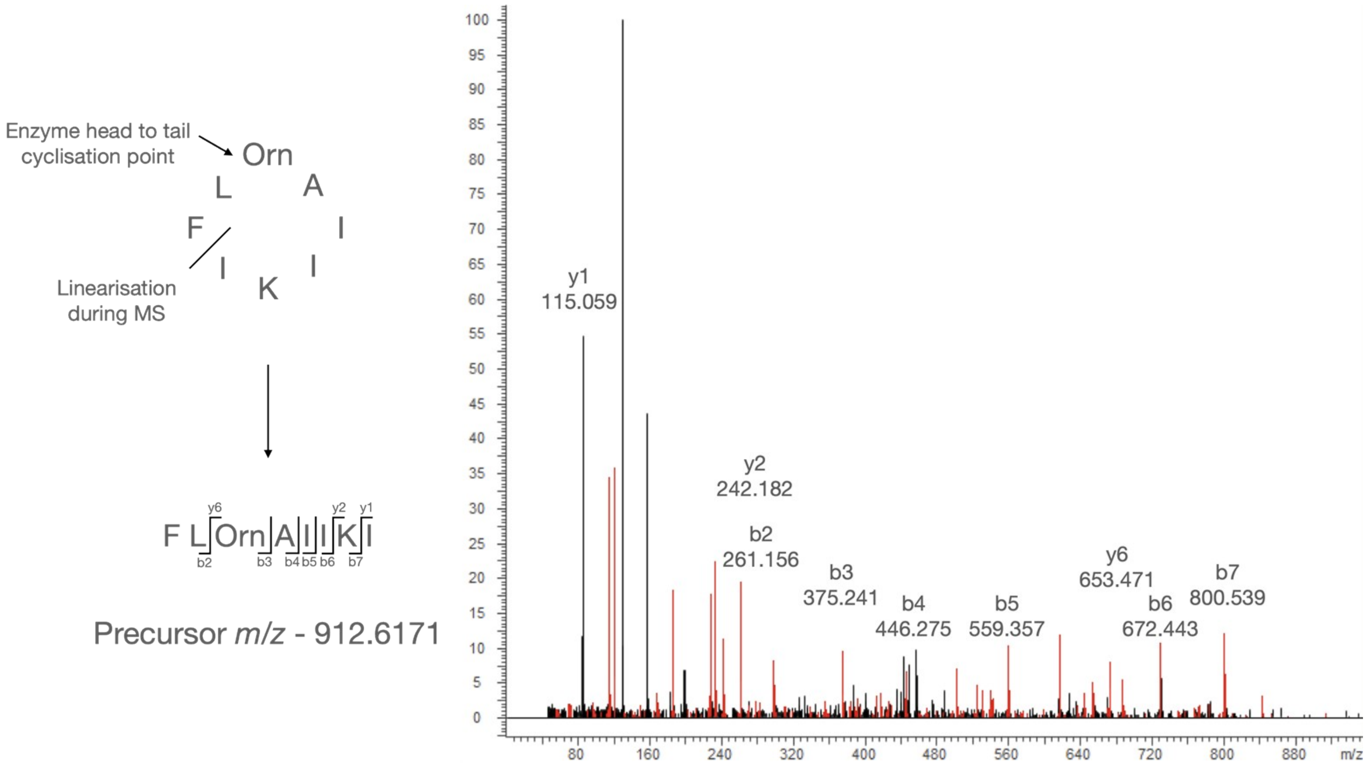

Fig. S36. MSMS analysis of fragment ions produced during assays of SNAC-Orn-surugamide A with SurE. The peaks corresponding to each fragment ion are labelled with the representative *m/z* value. The nature of the precursor peptide is shown beside the mass spectrum in each case.

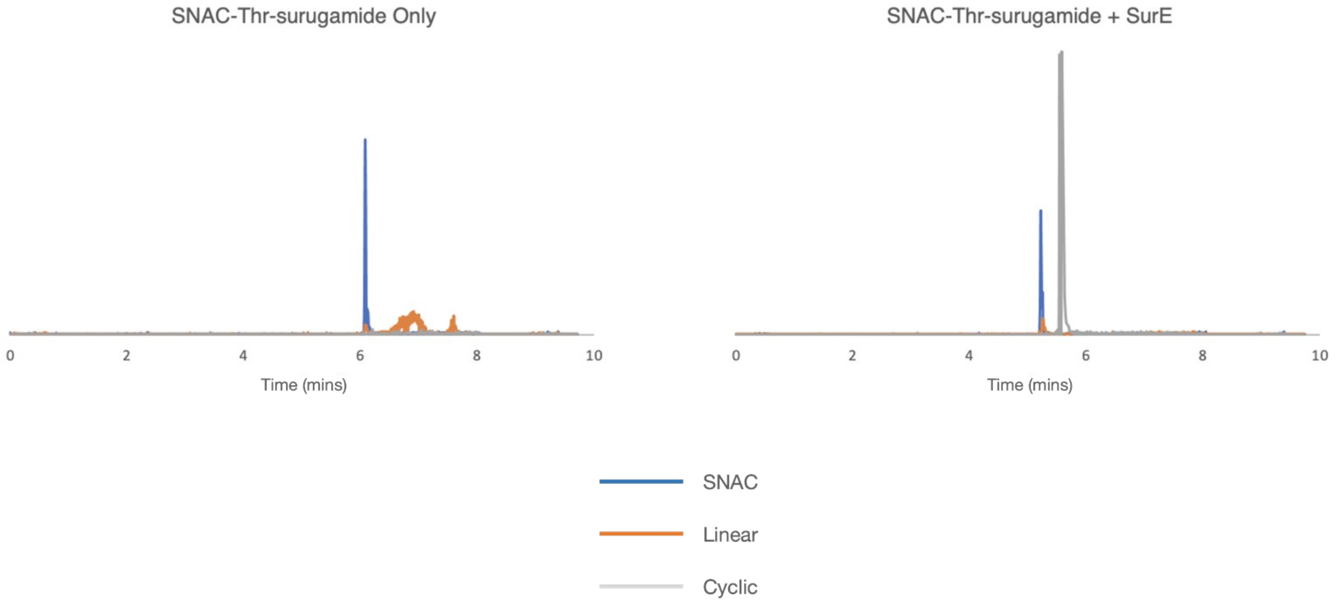

Fig. S37. LC-HRMS analysis of assays involving SNAC-Thr-surugamide A (the N-terminal Thr variant of SNAC-surugamide A) with (right) or without (left) SurE. Extracted ion chromatograms corresponding to the $[M+H]^+$ and $[M+Na]^+$ ions of the complete SNAC form of the peptide (blue), as well as the linear (orange) and cyclic (grey) forms are displayed. The *x* axis is displayed in units of time (mins) and the *y* axis was set to 7×10^4 for each of the chromatograms.

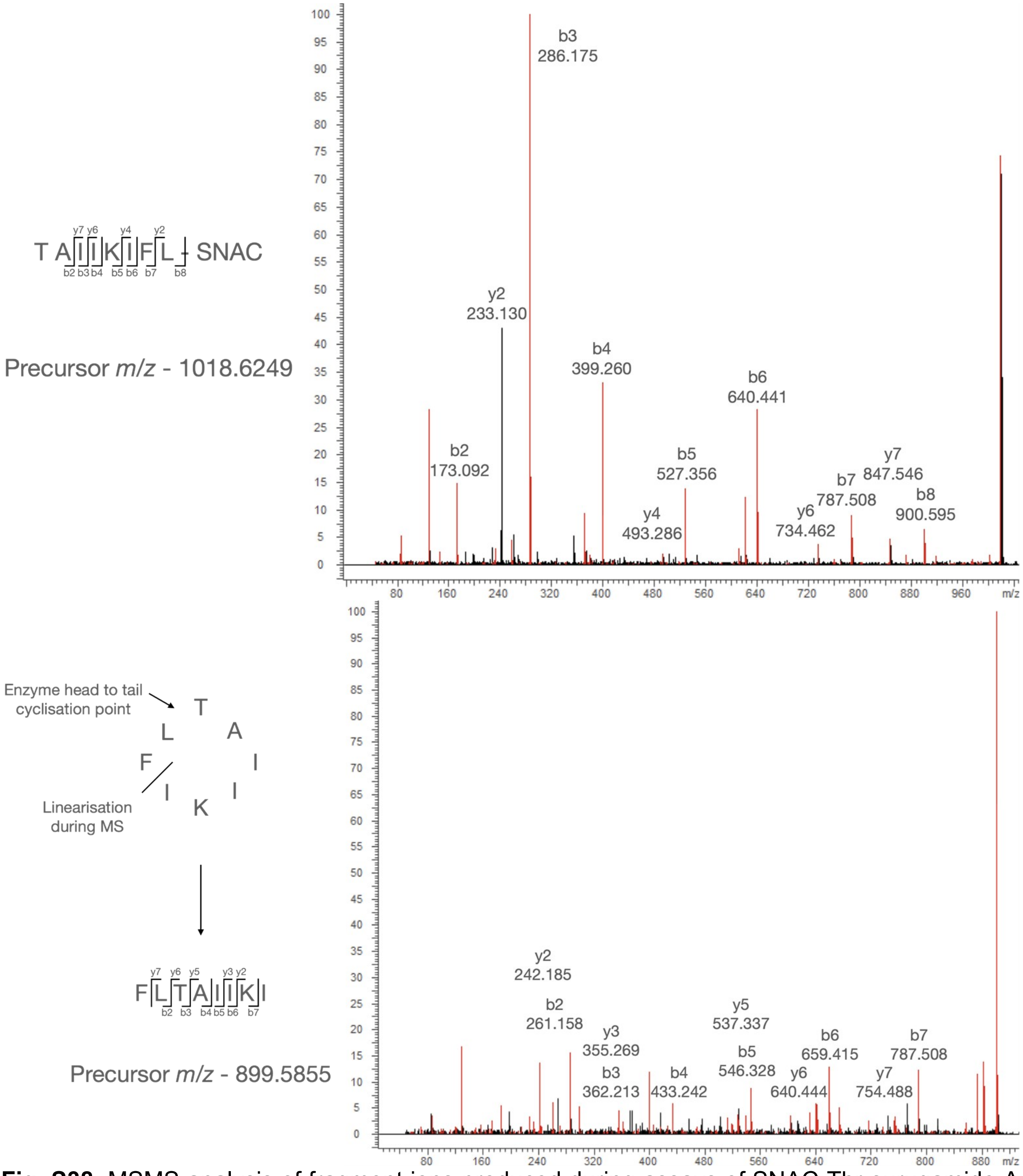

Fig. S38. MSMS analysis of fragment ions produced during assays of SNAC-Thr-surugamide A with SurE. The peaks corresponding to each fragment ion are labelled with the representative *m/z* value. The nature of the precursor peptide is shown beside the mass spectrum in each case.

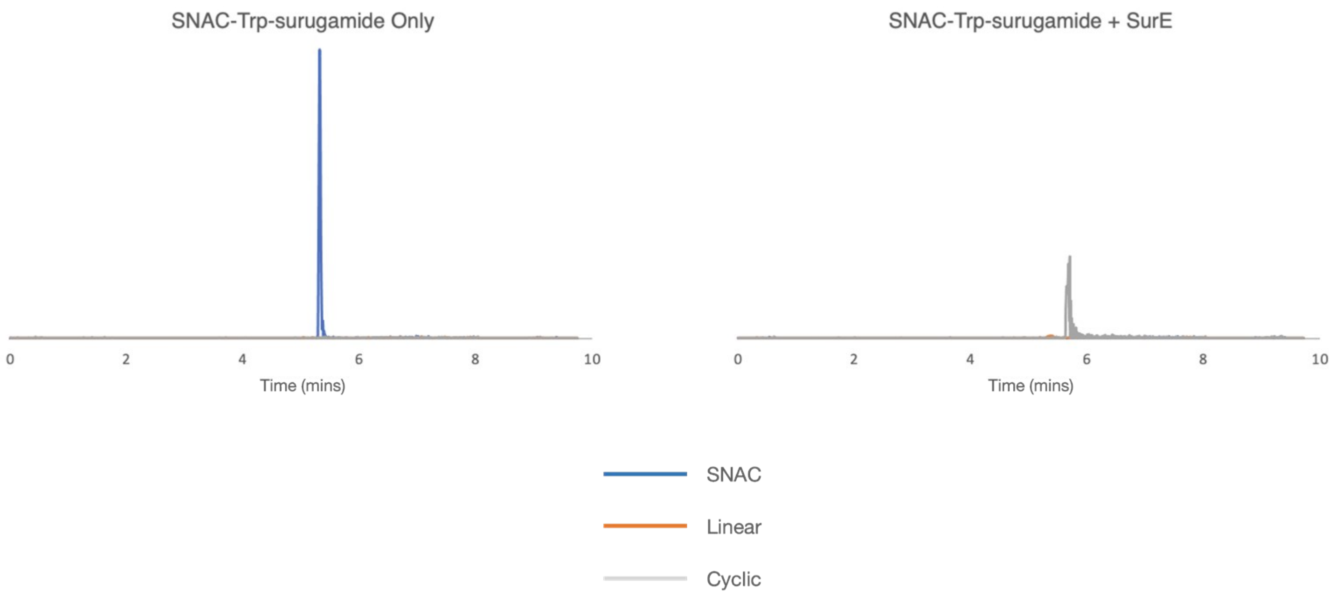

Fig. S39. LC-HRMS analysis of assays involving SNAC-Trp-surugamide A (the N-terminal Trp variant of SNAC-surugamide A) with (right) or without (left) SurE. Extracted ion chromatograms corresponding to the $[M+H]^+$ and $[M+Na]^+$ ions of the complete SNAC form of the peptide (blue), as well as the linear (orange) and cyclic (grey) forms are displayed. The *x* axis is displayed in units of time (mins) and the *y* axis was set to 8×10^4 for each of the chromatograms.

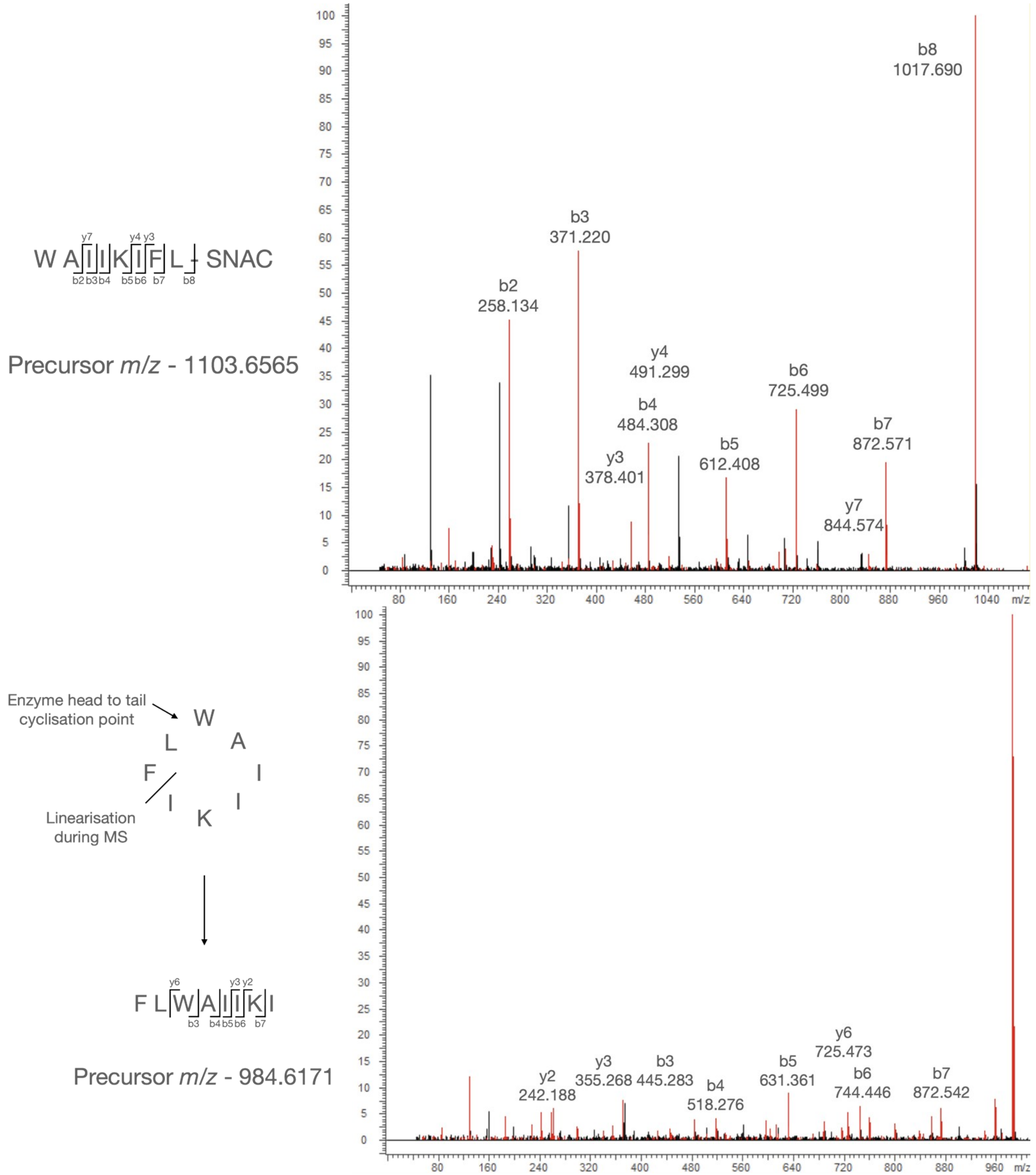

Fig. S40. MSMS analysis of fragment ions produced during assays of SNAC-Trp-surugamide A with SurE. The peaks corresponding to each fragment ion are labelled with the representative *m/z* value. The nature of the precursor peptide is shown beside the mass spectrum in each case.

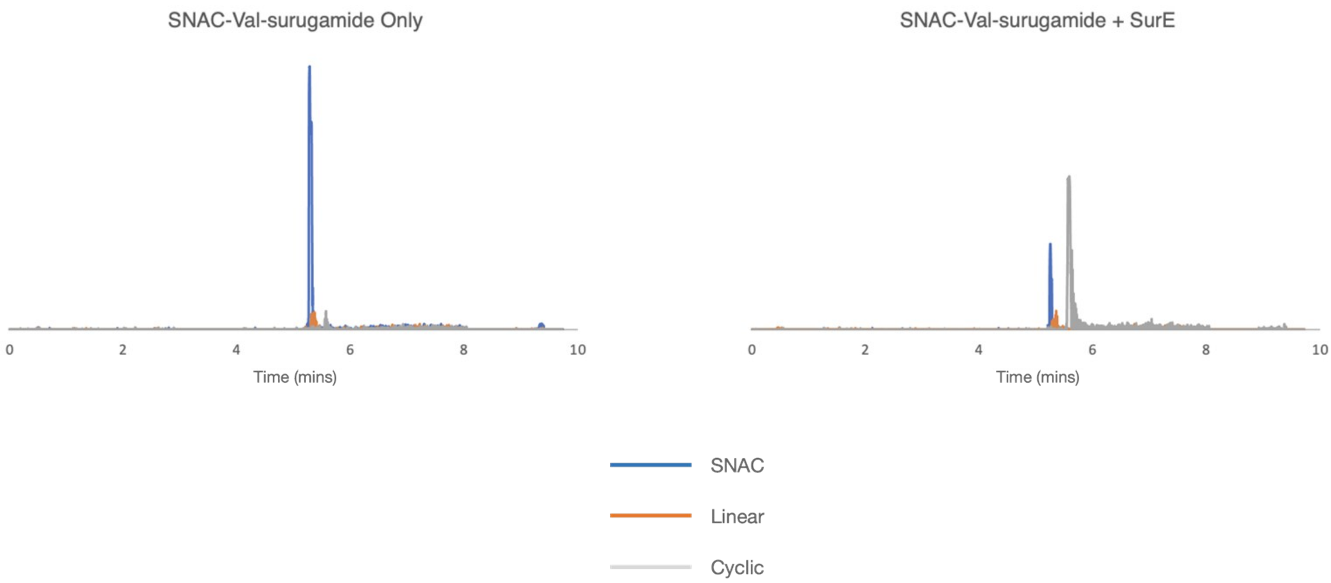

Fig. S41. LC-HRMS analysis of assays involving SNAC-Val-surugamide A (the N-terminal Val variant of SNAC-surugamide A) with (right) or without (left) SurE. Extracted ion chromatograms corresponding to the $[M+H]^+$ and $[M+Na]^+$ ions of the complete SNAC form of the peptide (blue), as well as the linear (orange) and cyclic (grey) forms are displayed. The *x* axis is displayed in units of time (mins) and the *y* axis was set to 4×10^4 for each of the chromatograms.

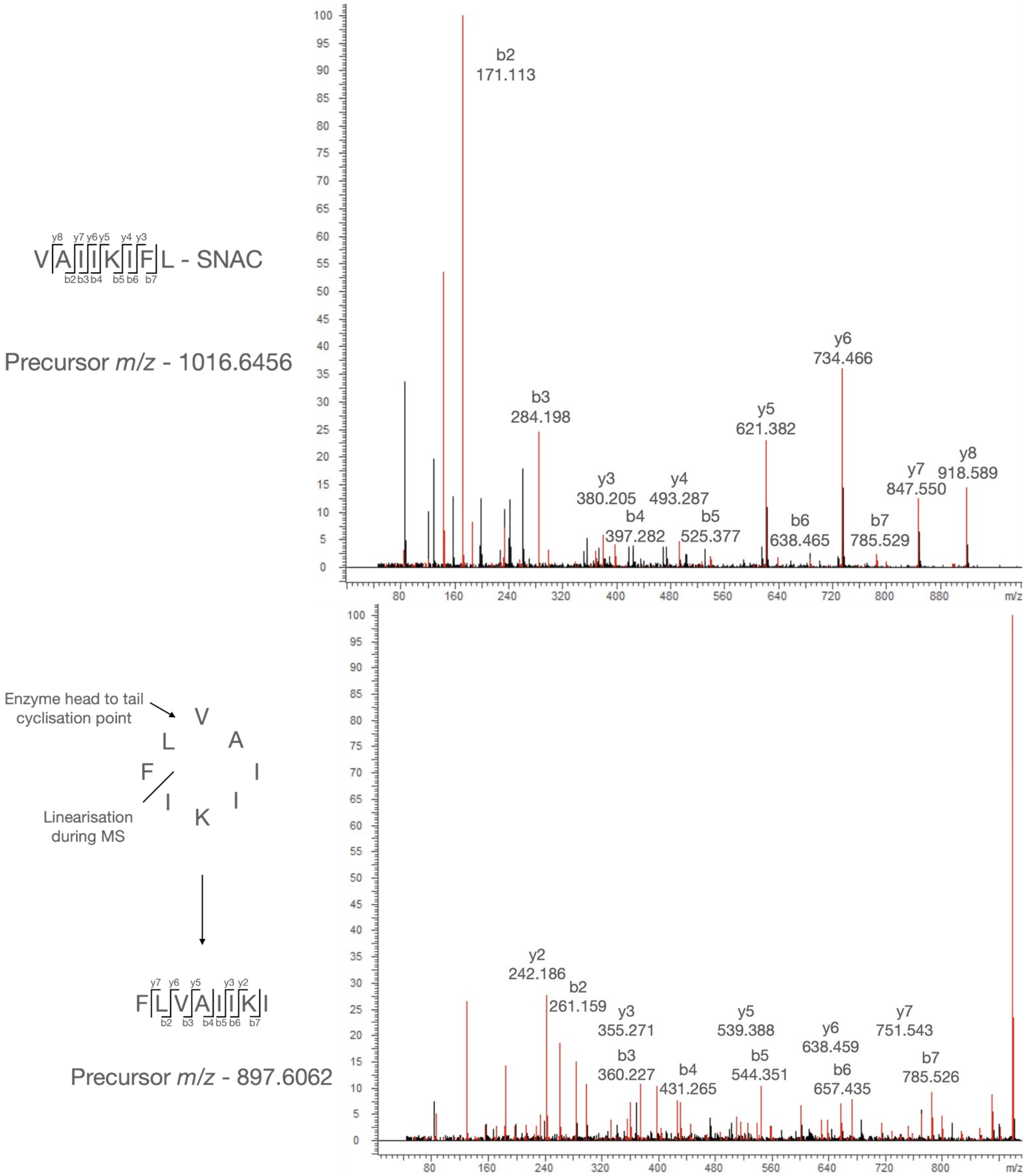

Fig. S42. MSMS analysis of fragment ions produced during assays of SNAC-Val-surugamide A with SurE. The peaks corresponding to each fragment ion are labelled with the representative *m/z* value. The nature of the precursor peptide is shown beside the mass spectrum in each case.

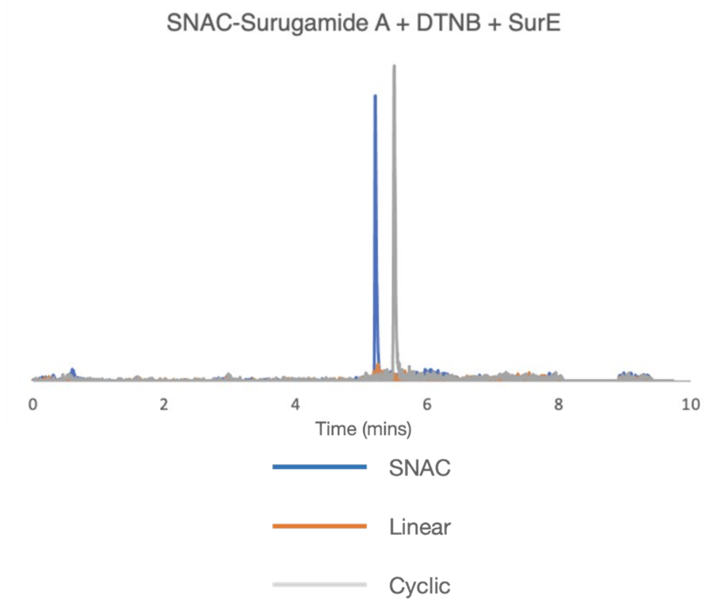

Fig. S43. LC-HRMS analysis of assays involving SNAC-surugamide A with SurE, in the presence of the colorimetric substrate DTNB. Extracted ion chromatograms corresponding to the $[M+H]^+$ and $[M+Na]^+$ ions of the complete SNAC form of the peptide (blue), as well as the linear (orange) and cyclic (grey) forms are displayed. The *x* axis is displayed in units of time (mins) and the *y* axis was set to 4×10^4 for each of the chromatograms.

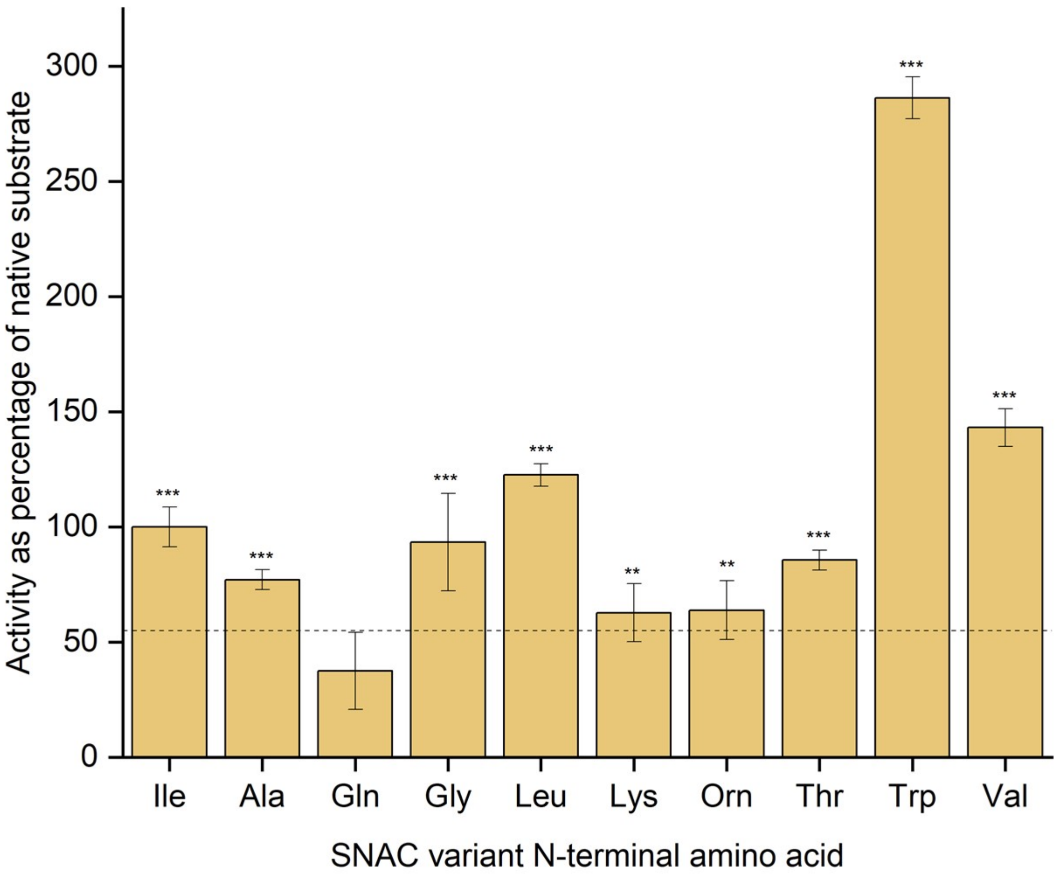

Fig. S44. Analysis of SurE enzymatic activity on various SNAC-substrates, with varying N-terminal amino acids as indicated by the x-axis. Assays were carried out in triplicate using a substrate concentration of 100 μM. The mean initial rate as a percentage of the mean rate observed for the WT surugamide A peptide is displayed for each substrate, with error bars signifying the standard error of the mean (S.E.M.). The horizontal line at y = 55 represents a threshold above which mean initial rate yields a significant p value (assuming zero error in any measurements and 95% confidence limit).

**Substrate** (Denoted by N-terminal amino acid)	Mean initial rate at 100 μM substrate concentration (μM/min ± S.E.M.)	Mean initial rate at 200 μM substrate concentration (μM/min ± S.E.M.)
**Ile**	1.56 ± 0.13	1.98 ± 0.15
**Ala**	1.19 ± 0.05	2.02 ± 0.53
**Gln**	0.58 ± 0.09	0.77 ± 0.15
**Gly**	1.45 ± 0.30	2.31 ± 0.52
**Leu**	1.91 ± 0.10	3.74 ± 0.28
**Lys**	0.98 ± 0.12	1.37 ± 0.12
**Orn**	0.99 ± 0.12	2.01 ± 0.25
**Thr**	1.33 ± 0.06	1.98 ± 0.36
**Trp**	4.45 ± 0.40	7.11 ± 0.72
**Val**	2.23 ± 0.18	3.43 ± 0.54

Table S1. Calculated mean initial rates of activity of SurE with SNAC-substrates containing the noted N-terminal amino acid. The mean (with standard error) of triplicate experiments is shown for each substrate at the two substrate concentrations tested.

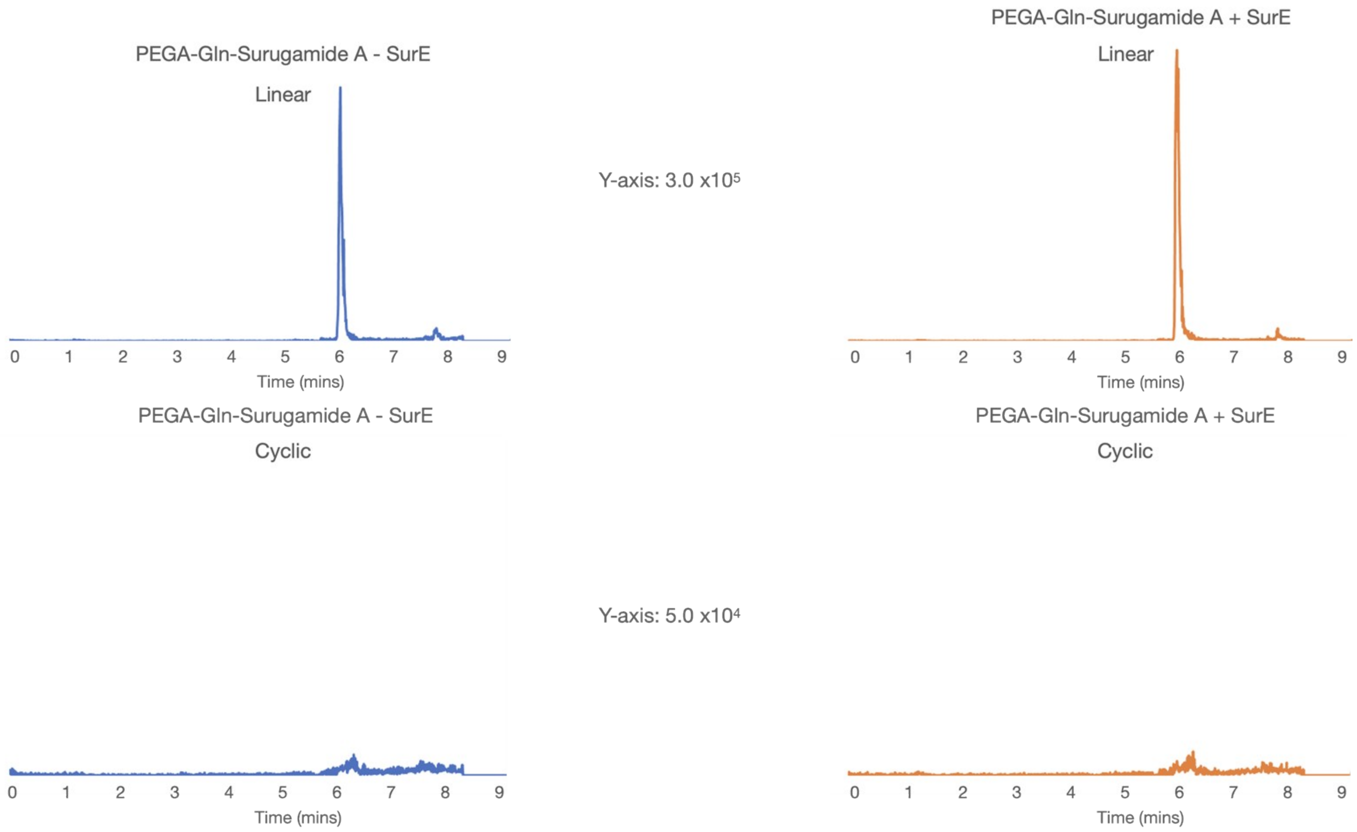

Fig. S45. LC-HRMS analysis of larger scale assays involving PEGA-Gln-surugamide A with SurE. Extracted ion chromatograms corresponding to the $[M+H]^+$ and $[M+Na]^+$ ions of the linear form of the peptide (top), as well as the cyclic (bottom) form are displayed. The *x* axis is displayed in units of time (mins) and the *y* axis scales for the linear and cyclic chromatograms are displayed accordingly.

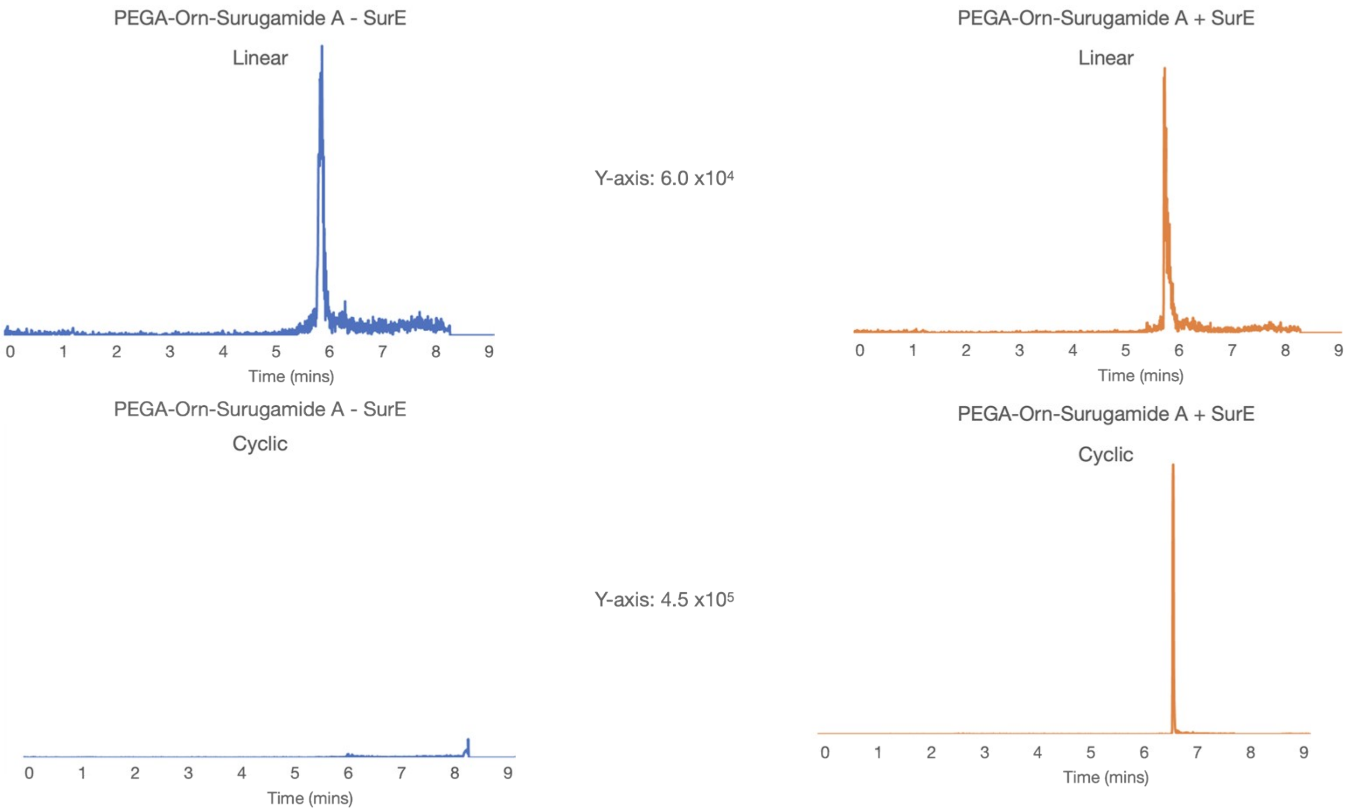

Fig. S46. LC-HRMS analysis of larger assays involving PEGA-Orn-surugamide A with SurE. Extracted ion chromatograms corresponding to the $[M+H]^+$ and $[M+Na]^+$ ions of the linear form of the peptide (top), as well as the cyclic (bottom) form are displayed. The *x* axis is displayed in units of time (mins) and the *y* axis scales for the linear and cyclic chromatograms are displayed accordingly.

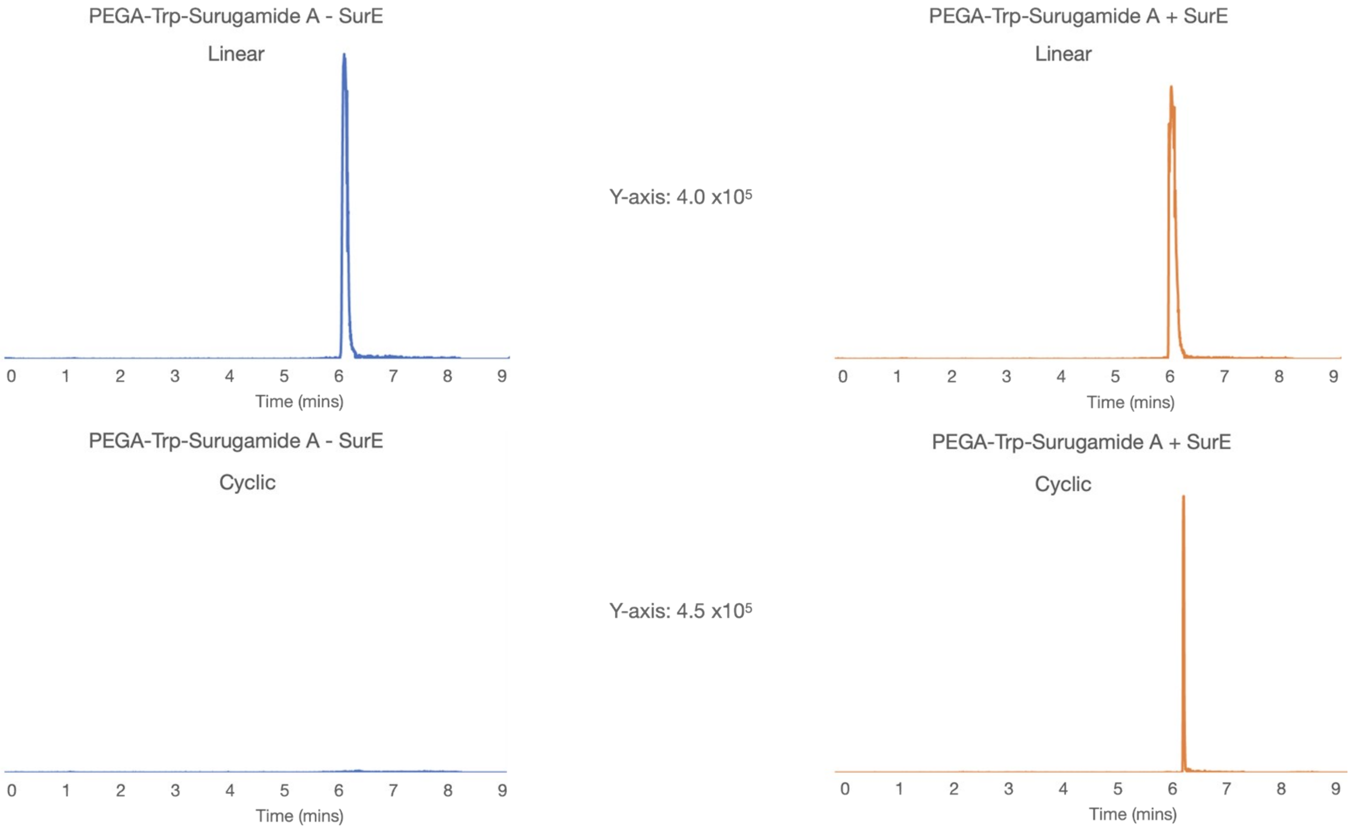

Fig. S47. LC-HRMS analysis of larger scale assays involving PEGA-Trp-surugamide A with SurE. Extracted ion chromatograms corresponding to the $[M+H]^+$ and $[M+Na]^+$ ions of the linear form of the peptide (top), as well as the cyclic (bottom) form are displayed. The *x* axis is displayed in units of time (mins) and the *y* axis scales for the linear and cyclic chromatograms are displayed accordingly.

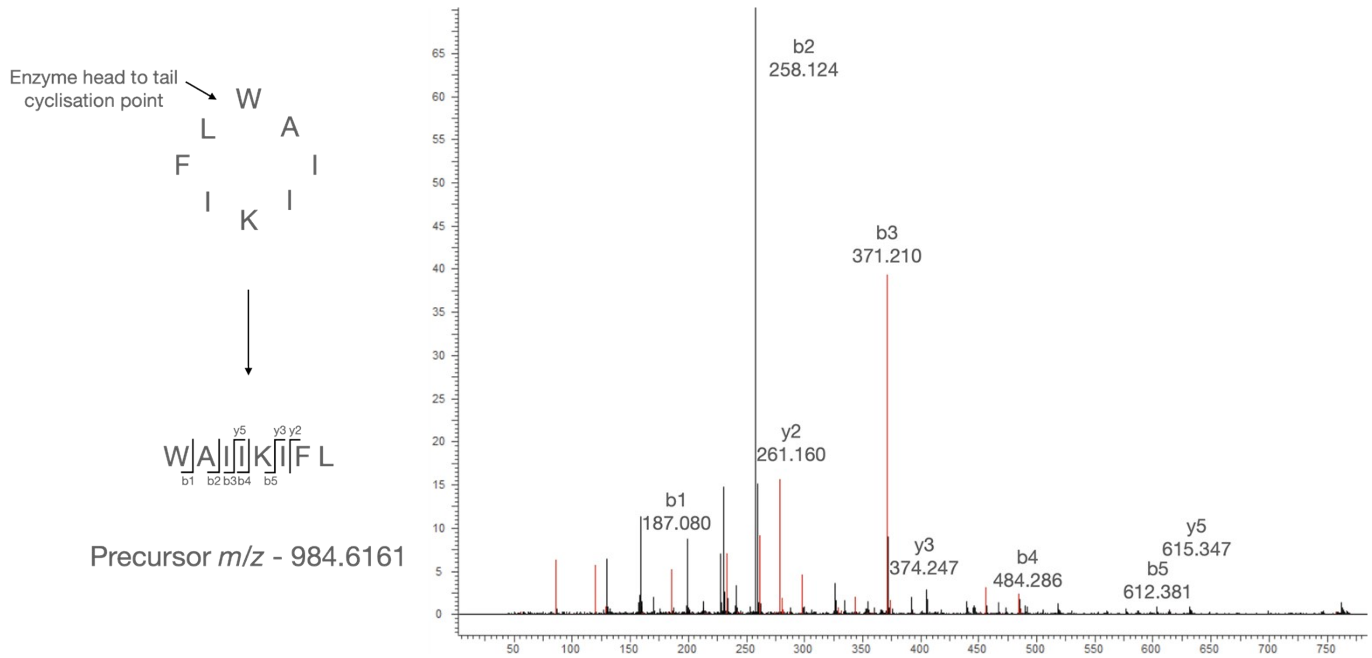

Fig. S48. MSMS analysis of fragment ions produced from cyclic Trp-surugamide A, the product of the PEGA-Trp-surugamide A reaction with SurE. The peaks corresponding to each fragment ion are labelled with the representative *m/z* value. The nature of the precursor peptide is shown beside the mass spectrum.
